# Effective Stimulation Type and Waveform for Force Control of the Motor Unit System: Implications for Intraspinal Microstimulation

**DOI:** 10.3389/fnins.2021.645984

**Published:** 2021-06-28

**Authors:** Hojeong Kim, Youngchang Ju

**Affiliations:** ^1^Division of Biotechnology, DGIST, Daegu, South Korea; ^2^Department of Brain and Cognitive Sciences, DGIST, Daegu, South Korea

**Keywords:** stimulation waveform, force control, motor unit, neuromodulation, intraspinal microstimulation, stimulation type

## Abstract

The input–output properties of spinal motoneurons and muscle fibers comprising motor units are highly non-linear. The goal of this study was to investigate the stimulation type (continuous versus discrete) and waveform (linear versus non-linear) controlling force production at the motor unit level under intraspinal microstimulation. We constructed a physiological model of the motor unit with computer software enabling virtual experiments on single motor units under a wide range of input conditions, including intracellular and synaptic stimulation of the motoneuron and variation in the muscle length under neuromodulatory inputs originating from the brainstem. Continuous current intensity and impulse current frequency waveforms were inversely estimated such that the motor unit could linearly develop and relax the muscle force within a broad range of contraction speeds and levels during isometric contraction at various muscle lengths. Under both continuous and discrete stimulation, the stimulation waveform non-linearity increased with increasing speed and level of force production and with decreasing muscle length. Only discrete stimulation could control force relaxation at all muscle lengths. In contrast, continuous stimulation could not control force relaxation at high contraction levels in shorter-than-optimal muscles due to persistent inward current saturation on the motoneuron dendrites. These results indicate that non-linear adjustment of the stimulation waveform is more effective in regard to varying the force profile and muscle length and that the discrete stimulation protocol is a more robust approach for designing stimulation patterns aimed at neural interfaces for precise movement control under pathological conditions.

## Introduction

Recent advances in neural interface technology have allowed the direct modulation of nervous system functions by injecting currents into specific compartments of individual neurons (Holsheimer, 1998; Radivojevic et al., 2016). In regard to neuromuscular systems, individual motor units, consisting of a single motoneuron and its innervating muscle fibers (i.e., the muscle unit), could represent a target modulated at the spinal cord to accurately evoke proper movements (Wagner et al., 2018). However, the prediction of stimulation patterns and their effects on force control have remained difficult, mainly due to the non-linearities inherited in the fundamental elements comprising neuromuscular systems (Heckman and Enoka, 2012).

The input–output relationship of spinal motoneurons is highly non-linear in various species, including rats, mice, cats, and turtles (Heckman et al., 2003). The firing mode of the motoneuron may transition from quiescent to regular firing or from low- to high-frequency firing in response to brief excitatory current injection at the soma, thereby revealing the occurrence of bistability (Hounsgaard et al., 1988). While slowly increasing and decreasing current injection at the soma, low-threshold motoneurons (presumably slow-type motoneurons) tend to exhibit notable counterclockwise hysteresis and a self-sustaining firing behavior below the firing initiation threshold in the descending stimulation phase, whereas high-threshold motoneurons (presumably fast-type motoneurons) tend to exhibit slight clockwise hysteresis under the absence of self-sustaining firing behavior (Lee and Heckman, 1998b). The underlying mechanism of this non-linear input–output relationship has been suggested as the spatiotemporal interaction between action potential-producing membrane mechanisms at the soma and plateau potential-generating calcium channels in dendritic areas (i.e., 300–800 μm from the soma) (Kim et al., 2014). These dendritic calcium channels (presumably L-type Ca_v_1.3 channels) are actively involved through monoaminergic neuromodulation due to the brainstem regarding normal motor behavior and through endogenous monoamines in regard to spinal cord injury (Heckmann et al., 2005).

The input–output relationship of muscle fibers has also been demonstrated to be highly non-linear in frogs, rats, and cats (Mrowczynski et al., 2006). Compared to its low frequency, a greater muscle force is produced in response to high-frequency current stimulation, resulting in a sigmoid curve of the relationship between the stimulation frequency and force output. In addition, the muscle force is maximized at the optimal muscle length over smaller and larger muscle lengths, thereby revealing a bell-shaped relationship between the muscle length and force output during isometric contraction (Winters et al., 2011). This non-linear relationship is attributable to the complex interactions between calcium dynamics, cross-bridge formation, and length variation in the sarcoplasm of muscle fibers (Kim et al., 2015). In summary, the dynamics of muscle activation are non-linearly related to both the stimulation frequency and muscle length and greatly decrease under shorter-than-optimal lengths at the physiological stimulation frequency (i.e., <20 Hz) (Perreault et al., 2003).

Intraspinal microstimulation exhibits the capability of activating a specific set of neurons within the spinal cord to modulate muscle activity in different body parts, including the hindlimb (Mushahwar and Horch, 1998), forelimb (Sunshine et al., 2013), and respiratory system (Sunshine et al., 2018). Recently, the relationship between current stimulation and motoneuron firing has been investigated *via* the direct application of direct or pulsed current injection to the spinal cord. The intradural region of the spinal cord in mice has been targeted for direct current stimulation purposes to elucidate the influence of the stimulation polarity on the firing outputs of hindlimb motoneurons (Ahmed, 2016). Computational and imaging studies have further demonstrated that the asymmetric waveform of a biphasic stimulus current pulse may enhance the target selectivity, and the stimulus frequency may alter the neuronal output (McIntyre and Grill, 2000; Wang et al., 2012). However, little is known regarding the stimulation pattern to produce desired force profiles *via* skeletal muscles at the motor unit level, which is the smallest element underlying all movements.

Here, we theoretically investigated the effective waveforms during intraspinal microstimulation to control the muscle force under two types of stimulation protocols, namely, continuous and discrete (or impulse) current stimulation protocols, during isometric muscle contraction within a full physiological range of the muscle length and force level. The present study focused on the direct activation of a spinal motoneuron with a microelectrode in close proximity to its initial segment and cell body. Extracellular microstimulation of other components, such as axons or dendrites, was not involved in the current analysis. We hypothesize (1) that non-linear stimulation waveforms are needed to elicit linear force generation at the motor unit level and (2) that the discrete current stimulation protocol more effectively prevents the non-linearities induced by the active channels in motoneuron dendrites, including muscle afferent inputs due to muscle length variation. Simulation analysis demonstrated the systematic stimulation waveform non-linearity depending on the stimulation type, muscle length, and force profile. These simulation results provide a basis for the design of stimulation patterns for neural interfaces to enhance motor precision control under pathological conditions.

## Materials and Methods

### Motor Unit Model

**FIGURE 1:**
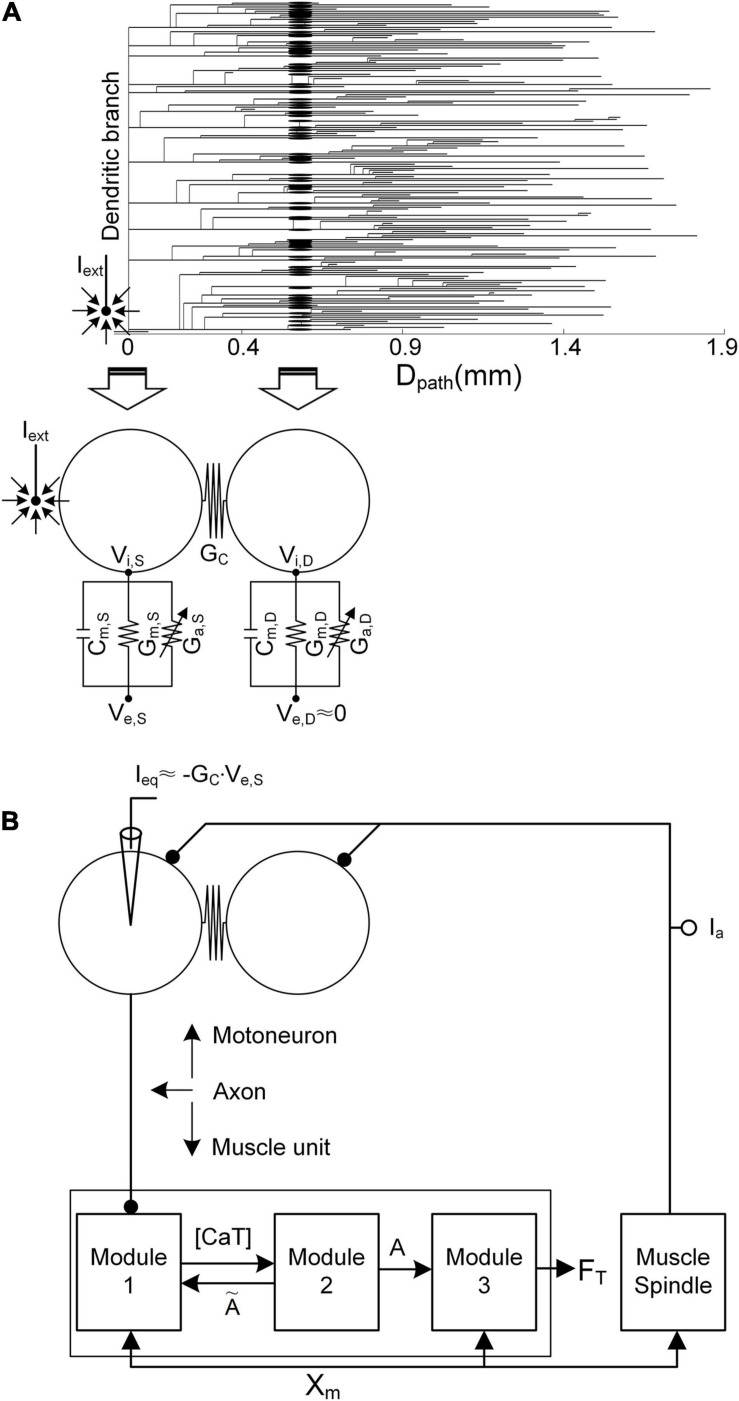
Simulation setup. **(A)** Reduction in the anatomical motoneuron (*top*, dendrogram) into a two-compartment framework (*bottom*) as a function of the path length (*D*_path_) from the center of the cell body (*D*_path_ = 0). The left arrow indicates the mapping of the soma/axonal hillock/initial segment of the anatomical model into the somatic compartment of the reduced model. The right arrow indicates the mapping of all points over the narrow band (a mean value of 0.6 mm) of the anatomical dendrites into the dendritic compartment of the reduced model. *C*_m_, *G*_m_, *G*_a_ *G*_C_, *V*_i_, and *V*_e_ indicate the membrane capacitance, leak conductance, voltage-gated conductance, coupling conductance, and intracellular and extracellular potentials, respectively. The subscripts S and D indicate the somatic and dendritic compartments, respectively. *I*_ext_, the current passing through the microelectrode, is located close to the initial segment and cell body. In the current analysis, *V*_e,S_ is determined by *I*_ext_, while the influence of *I*_ext_ on *V*_e,D_ is assumed to be negligible. **(B)** Model motor unit comprising the two-compartment motoneuron model and three-module muscle unit along with the muscle spindle afferent (*I*_a_). *I*_eq_ is the equivalent current intracellularly injected at the somatic compartment of the motoneuron to evoke the same transmembrane potential induced by *I*_ext_. [CaT], *A*, *F*_T_, and *X*_m_ indicate the concentration of calcium bound to troponin, the muscle activation level, the force output, and the muscle length, respectively.

Stimulation waveforms were investigated in regard to extracellular microstimulation with a sharp-tipped electrode near the initial segment and cell body of a spinal motoneuron (Figure 1). A motor unit model comprising a two-compartment motoneuron model and a three-module muscle unit model was physiologically constructed and simulated in Python-based Motor Unit Simulator (PyMUS) software, which was developed for virtual experiments on single motor units under a wide range of physiological conditions (Kim and Kim, 2018).

**FIGURE 2:**
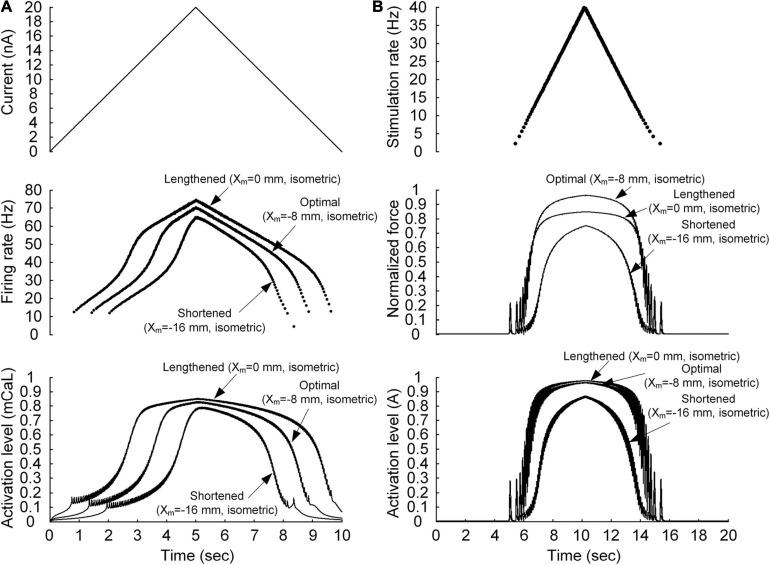
Non-linear behavior of the motoneuron **(A)** and motor unit **(B)**. **(A)** Current stimulation applied to the somatic compartment of the motoneuron model (*top*), instantaneous firing rate (*middle*), and dynamics of the PIC activation level in the motoneuron dendrite (*bottom*). The arrows indicate the responses at the three muscle lengths (minimal length with *X*_m_ = −16 mm, optimal length with *X*_m_ = −8 mm, and maximal length with *X*_m_ = 0 mm). **(B)** Frequency of impulse current stimulation applied to the muscle unit model (*top*), force normalized with the peak force at the optimal length (*middle*), and dynamics of the muscle activation level (*bottom*). The arrows indicate the responses at the three muscle lengths (minimal length with *X*_m_ = −16 mm, optimal length with *X*_m_ = −8 mm, and maximal length with *X*_m_ = 0 mm).

Briefly, in terms of the reduced motoneuron model, one compartment (referred to as the somatic compartment) represents the soma and axonal hillock/initial segment, and the other compartment (denoted as the dendritic compartment) represents the dendritic regions, including persistent inward current-generating voltage-gated channels (Booth et al., 1997). These two compartments are connected *via* the coupling conductance, representing the electrical distance between the soma and dendritic regions. Five passive parameters, including the specific membrane conductance and capacitance (i.e., *G*_m__,S_ and *C*_m__,S_ of the somatic compartment, *G*_m__,D_ and *C*_m__,D_ of the dendritic compartment, and G_C_ in regard to the coupling conductance between these two compartments), were analytically determined to capture five electrotonical properties, including the somatic input resistance (i.e., *R*_N__,S_), system time constant (i.e., *τ_m_*), and three voltage attenuation factors between the soma and all dendritic sites separated by a similar path length (i.e., *D*_path_) (i.e., ${\text{VA}}_{\text{SD}}^{\text{DC}}$ and ${\text{VA}}_{\text{SD}}^{\text{AC}}$ describing soma-to-dendrite propagation of direct and alternative currents and ${\text{VA}}_{\text{DS}}^{\text{DC}}$ describing dendrite-to-soma propagation of a direct current). Action and plateau potentials were generated in the somatic and dendritic compartments *via* the incorporation of Hodgkin–Huxley-style active channels [i.e., *G*_Naf_, *G*_Nap_, *G*_Kdr_, *G*_K(Ca)_, and *G*_Can_, with dynamic changes in the calcium reversal potential of the soma, and G_Cal_, with a constant calcium reversal potential of the dendrites]. The passive parameter values were first determined to capture the passive properties [*R*_N__,S_ = 1.29 MΩ, *τ_m_* = 7.2 ms, ${\text{VA}}_{\text{SD}}^{\text{DC}}$(*D*_path_ = 0.6 mm) = 0.76, ${\text{VA}}_{\text{SD}}^{\text{AC}}$(*D*_path_ = 0.6 mm) = 0.27, and ${\text{VA}}_{\text{DS}}^{\text{DC}}$(*D*_path_ = 0.6 mm) = 0.75] measured at the soma and dendrites as previously reported in Zengel et al. (1985); Spruston et al. (1994), and Kim et al. (2014). Then, the active parameters were determined to replicate the active properties (spike height = 92.3 mV, rheobase current = 10.5 nA, afterhyperpolarization duration = 98.5 ms, and depth = 3.1 mV under passive dendrites, and effective persistent inward current = −22 nA under active dendrites) obtained from the cell body as previously mentioned in Zengel et al. (1985); Hochman and McCrea (1994), and Hornby et al. (2002). The voltage–current (i.e., VI) and current–voltage (i.e., IV) properties of the model motoneuron were validated *via* a comparison to those of an anatomically reconstructed motoneuron model that reproduces non-linear behaviors observed from *in vivo* cat α-motoneurons with low thresholds during voltage- and current-clamping at the cell body (Kim et al., 2014) (please refer to Figure 2A for non-linear behavior of the model motoneuron).

Regarding the modular muscle unit model, the muscle force was produced through three physiological procedures in response to action potentials originating from the motoneuron. Module 1 transforms neural signals into dynamics of the calcium concentration (i.e., Ca_SP_) in the sarcoplasm, including calcium release and uptake of the sarcoplasmic reticulum, calcium-buffering proteins, and calcium-bound troponin complex. Module 2 then transforms the concentration dynamics of calcium-bound troponin (i.e., Ca_SP_T) into the degree of cross-bridge formation, representing muscle activation dynamics (i.e., A). Finally, module 3 transforms the above muscle activation dynamics into force based on Hill-type muscle–tendon mechanics, reflecting length–and velocity–tension relationships. Model parameter values were determined for each module to reproduce the force generation process. These values reflect sarcoplasmic calcium dynamics (Westerblad and Allen, 1994) in module 1, calcium–force relationship (Shames et al., 1996) in module 2, length–and velocity–tension properties (Scott et al., 1996) in module 3, and muscle activation dependence on muscle length variation (Sandercock and Heckman, 1997) in modules 1 and 3. The input–output properties of the model muscle unit have been validated through a comparison to those of adult cat soleus muscles within a full physiological range of stimulation rates (i.e., 1–100 Hz) and muscle lengths (i.e., approximately -16 to 0 mm) during isometric, isokinetic, and dynamic contractions (Kim et al., 2015) (please refer to Figure 2B for non-linear behavior of the model muscle unit).

The axonal nerve coupling the motoneuron with the muscle unit was modeled *via* a single parameter representing the delay time (∼10 ms) required for the transmission of action potentials from the motoneuron to the muscle unit, assuming perfect action potential transmission from the motoneuron to the muscle unit (Kim, 2017). The overall input–output properties of the model motor unit were validated by comparison to those of a physiologically reconstructed model for an adult cat slow-type motor unit in the previous study [please refer to Figures 8, 9 in Kim and Kim (2018) for non-linear behavior of the model motor unit]. The system equations and parameter values used for this study are presented as supplementary material (Supplementary File 1).

### Stimulation Protocols

The non-linearity of the input–output relationship was investigated separately for the motoneuron and muscle unit. In regard to the motoneuron, a linearly ascending and descending current with a peak value of 20 nA over 10 s was injected at the soma of the motoneuron (as shown in Figure 2A). In regard to the muscle unit, a train of current impulses was applied such that the stimulation frequency increased and decreased according to a triangular shape with a peak value of 40 Hz over 10 s (see Figure 2B). The peak stimulation frequency was selected to ensure that the model muscle unit produced the maximal force during isometric contraction at the optimal muscle length.

In the present study, stimulation waveforms were investigated under extracellular motoneuron microstimulation with a sharp-tipped electrode near the initial segment and cell body (Figure 1). Under these conditions, the extracellular potential (i.e., *V*_e,S_) at the soma is determined by the current (i.e., *I*_ext_) passing through the microelectrode, whereas the extracellular potential (i.e., *V*_e,D_) across the dendritic area (i.e., *D*_path_ > 0.6 mm) distal to the soma tends to not depend on *I*_ext_ (McIntyre and Grill, 2002). The variation in transmembrane potential (i.e., *V*_S_) at the soma due to *I*_ext_ was simulated *via* intracellular injection of the equivalent current (i.e., *I*_eq_) at the soma, as proposed in a previous study (Warman et al., 1992). With the application of Kirchhoff’s current law to the somatic compartment of the two-compartment model in addition to approximation of *V*_e,D_ as zero under passive membrane conditions, *I*_eq_ can be derived as the following analytical form:

$$\begin{array}{c} I_{\text{eq}}=C_{m,\text{S}}\cdot \text{d}V_{\text{S}}/\text{d}t+G_{m,\text{S}}\cdot V_{\text{S}}+G_{\text{C}}\cdot V_{\text{S}}=-G_{\text{C}}\cdot V_{e,S} \end{array}$$

Where *V*_S_ is the potential difference between the intracellular (*V*_i,S_) and extracellular sides (*V*_e,S_) of the somatic compartment in the two-compartment neuron model.

Linear force production of the motor unit was induced considering two types (continuous and discrete) of current stimulation (i.e., *I*_eq_) in the somatic compartment of the motoneuron model over 10 s. The shape and amplitude of continuous current stimulation were first determined with a linear function in a piecewise manner so that the force linearly increased and decreased at the various speeds over 10 s. Then, piecewise linear functions were fitted with a continuous function ensuring linear force production by the motor unit (please refer to Table 1). In the case of discrete current stimulation, the timing and amplitude of the current pulses were adjusted such that the discrete current simulation process reproduced the temporal evolution of force production induced under continuous current stimulation condition. In this study, the amplitude of the current pulses indicated the minimal current intensity yielding a desired force profile. The width of the current pulse was set to 0.5 ms, ensuring a one-to-one stimulus-firing reaction at the stimulus amplitude threshold. To compare the different states between the various muscle lengths, all muscle forces induced under current stimulation in the somatic compartment of the motoneuron model were normalized based on the maximum force (i.e., P_0_) actively produced by the model motor unit during isometric contraction at the optimal muscle length. In the present study, the simulation data are not shown when the model motor unit could not produce the desired peak force (i.e., 20–100% of P_0_).

**TABLE 1:** Piecewise equations for the time courses of continuous current stimulation (i.e., *I*_eq_) presented in Figures 3, 5, 7.

Muscle length	Target force level	Figure 3	Figure 5	Figure 7
X_m_ = −16 mm	80% P_0_	∙ 0 < *t* ≤ 1,000: *I*_eq_ = 7.015⋅10^–3^ *t* ∙ 1,000 < *t* ≤ 4,950 *I*_eq_ = 28.99⋅sin[7.766⋅10^–4^⋅(*t* − 1,000) − 0.318] + 123.5⋅sin[1.445⋅10^–3^⋅(*t* – 1,000) + 1.768] + 105.7⋅sin [1.502⋅10^–3^⋅(*t* – 1,000) + 4.822] + 0.042 ∙ 4,950 < *t* ≤ 5,350: *I*_eq_ = −0.04⋅(*t* – 4,950) + 23.153 ∙ 5,350 < *t* ≤ 5,600: *I*_eq_ = 7.157 ∙ 5,600 < *t* ≤ 5,650: *I*_eq_ = 0.04⋅(*t* – 5,600) + 7.197 ∙ 5,650 < *t* ≤ 5,700: *I*_eq_ = −0.04⋅(*t* – 5,650) + 9.193 ∙ 5,700 < *t* ≤ 5,750: *I*_eq_ = 0.06⋅(*t* – 5,700) + 7.157 ∙ 5,750 < *t* ≤ 5,800: *I*_eq_ = −0.04⋅(*t* – 5,750) + 10.153 ∙ 5,800 < *t* ≤ 8,400: *I*_eq_ = 28.99⋅sin[7.766⋅10^–3^⋅(8,400 − *t*) − 0.318] + 123.5⋅sin [1.445⋅10^–3^⋅(8,400 − *t*) + 1.768] + 105.7⋅sin [1.502⋅10^–3^⋅(8,400 − *t*) + 4.822] + 0.042 ∙ 8,400 < *t* ≤ 9,400: *I*_eq_ = 7.015⋅10^–3^⋅(9,400–*t*) ∙ 9,400 < *t* ≤ 10,000: *I*_eq_ = 0	∙ 0 < *t* ≤ 5,000: *I*_eq_ = 10.81⋅exp[4.379⋅10^–5^⋅(2⋅*t* – 1,250.1)] + 3.544⋅10^–3^⋅exp [1.008⋅10^–3^⋅(2⋅*t* – 1,250.1)] ∙ 5,000 < *t* ≤ 10,000: *I*_eq_ = 10.81⋅exp{4.379⋅10^–5^⋅[2⋅(10,000 − *t*) – 1,250.1]} + 3.544⋅10^–3^⋅exp {1.008⋅10^–3^⋅[2⋅(10,000 − *t*) – 1,250.1]}	
	60% P_0_	∙ 0 < *t* ≤ 500: *I*_eq_ = 1.403⋅10^–2^⋅*t* ∙ 500 < *t* ≤ 1,380: *I*_eq_ = 7.015 ∙ 1,380 < *t* ≤ 4,950: *I*_eq_ = 28.99⋅sin[5.726⋅10^–4^⋅(*t* – 1,380) − 0.318] + 123.5⋅sin [1.064⋅10^–3^⋅(*t* – 1,380) + 1.768] + 105.7⋅sin [1.108⋅10^–3^⋅(*t* – 1,380) + 4.822] + 0.042 ∙ 4,950 < *t* ≤ 8,520: *I*_eq_ = 28.99⋅sin[5.726⋅10^–4^⋅(8,520 − *t*) − 0.318] + 123.5⋅sin[1.064⋅10^–3^⋅(8,520 − *t*) + 1.768] + 105.7⋅sin[1.108⋅10^–3^⋅(8,520 − *t*) + 4.822] + 0.042 ∙ 8,520 < *t* ≤ 8,700: *I*_eq_ = 7.015 ∙ 8,700 < *t* ≤ 9,200: *I*_eq_ = 1.403⋅10^–2^⋅(9,200 − *t*) ∙ 9,200 < *t* ≤ 10,000: *I*_eq_ = 0	∙ 0 < *t* ≤ 5,000: *I*_eq_ = 10.81⋅exp[4.379⋅10^–5^⋅(1.5⋅*t* – 1,250.1)] + 3.544⋅10^–3^⋅exp [1.008⋅10^–3^⋅(1.5⋅*t* – 1,250.1)] ∙ 5,000 < *t* ≤ 10,000: *I*_eq_ = 10.81⋅exp{4.379⋅10^–5^⋅ [1.5⋅(10,000 − *t*) – 1,250.1]} + 3.544⋅10^–3^⋅exp {1.008⋅10^–3^⋅[1.5⋅(10,000 − *t*) − 1,250.1]}	
	40% P_0_	∙ 0 < *t* ≤ 500: *I*_eq_ = 1.403⋅10^–2^⋅*t* ∙ 500 < *t* ≤ 1,380: *I*_eq_ = 7.015 ∙ 1,380 < *t* ≤ 4,950: *I*_eq_ = 28.99⋅sin[3.507⋅10^–4^⋅(*t* – 1,380) – 0.318] + 123.5⋅sin[0.652⋅10^–3^⋅(*t* – 1,380) + 1.768] + 105.7⋅sin[0.678⋅10^–3^⋅(*t* – 1,380) + 4.822] + 0.042 ∙ 4,950 < *t* ≤ 8,520: *I*_eq_ = 28.99⋅sin[3.507⋅10^–4^⋅(8,520 – *t*) − 0.318] + 123.5⋅sin[0.652⋅10^–3^⋅(8,520 – *t*) + 1.768] + 105.7⋅sin[0.678⋅10^–3^⋅(8,520 – *t*) + 4.822] + 0.042 ∙ 8,520 < t ≤ 8,700: *I*_eq_ = 7.015 ∙ 8,700 < *t* ≤ 9,200: *I*_eq_ = 1.403⋅10^–2^⋅(9,200 – *t*) ∙ 9,200 < *t* ≤ 10,000: *I*_eq_ = 0	∙ 0 < *t* ≤ 5,000: *I*_eq_ = 10.81⋅exp[4.379⋅10^–5^⋅(0.94⋅*t* – 1,250.1)] + 3.544⋅10^–3^⋅exp [1.008⋅10^–3^⋅(0.94⋅*t* – 1,250.1)] ∙ 5,000 < *t* ≤ 10,000: *I*_eq_ = 10.81⋅exp[4.379⋅10^–5^⋅(0.94⋅ (10,000 – *t*) – 1,250.1)] + 3.544⋅10^–3^⋅exp [1.008⋅10^–3^⋅(0.94⋅(10,000 – *t*) − 1,250.1)]	
	20% P_0_	∙ 0 < *t* ≤ 500: *I*_eq_ = 1.403⋅10^–2^⋅*t* ∙ 500 < *t* ≤ 1,380: *I*_eq_ = 7.015 ∙ 1,380 < *t* ≤ 4,950: *I*_eq_ = 28.99⋅sin[1.432⋅10^–4^⋅(*t* – 1,380) - 0.318] + 123.5⋅sin[0.266⋅10^–3^⋅(*t* – 1,380) + 1.768] + 105.7⋅sin [0.277⋅10^–3^⋅(*t* – 1,380) + 4.822] + 0.042 ∙ 4,950 < *t* ≤ 8,520: *I*_eq_ = 28.99⋅sin[1.432⋅10^–4^⋅(8,520 – *t*) – 0.318] + 123.5⋅sin[0.266⋅10^–3^⋅(8,520 – *t*) + 1.768] + 105.7⋅sin[0.277⋅10^–3^⋅(8,520 – *t*) + 4.822] + 0.042 ∙ 8,520 < t ≤ 8,700: *I*_eq_ = 7.015 ∙ 8,700 < *t* ≤ 9,200: *I*_eq_ = 1.403⋅10^–2^⋅(9,200 – *t*) ∙ 9,200 < *t* ≤ 10,000: *I*_eq_ = 0	∙ 0 < *t* ≤ 5,000: *I*_eq_ = 10.55 + 1.864⋅10^–4^⋅*t* ∙ 5,000 < *t* ≤ 10,000: *I*_eq_ = 10.55 + 1.864⋅10^–4^⋅ (10,000 – *t*)	
X_m_ = -8 mm	100% P_0_	∙ 0 < *t* ≤ 5,000: *I*_eq_ = 3.827⋅exp[4.696⋅10^–5^⋅(1.3⋅*t* + 3,500)] + 6.883⋅10^–7^⋅exp [1.545⋅10^–3^⋅(1.3⋅*t* + 3,500)] ∙ 5,000 < *t* ≤ 9,250: *I*_eq_ = 3.827⋅exp[4.696⋅10^–5^⋅ (1.3⋅(10,000 – *t*) + 3,500)] + 6.883⋅10^–7^⋅exp [1.545⋅10^–3^⋅(1.3⋅(10,000 – *t*) + 3,500)] ∙ 9,250 < *t* ≤ 10,000: *I*_eq_ = 0	∙ 0 < *t* ≤ 2,000: *I*_eq_ = 8.684⋅exp(-4.554⋅10^–6^⋅*t*) + 0.03⋅exp (1.03⋅10^–3^⋅*t*) ∙ 2,000 < *t* ≤ 5,000: *I*_eq_ = 8.543⋅exp(2.704⋅10^–5^⋅*t*) + 2.749⋅10^–5^⋅exp(2.772⋅10^–3^⋅*t*) - 0.18 ∙ 5,000 < *t* ≤ 8,000: *I*_eq_ = 8.543⋅exp[2.704⋅10^–5^⋅(10,000 – *t*)] + 2.749⋅10^–5^⋅exp[2.772⋅10^–3^⋅(10,000 – *t*)] - 0.18 ∙ 8,000 < *t* ≤ 10,000: *I*_eq_ = 8.684⋅exp[-4.554⋅10^–6^⋅(10,000 – *t*)] + 0.03⋅exp[1.03⋅10^–3^⋅(10,000 – *t*)]	∙ 0 < *t* ≤ 4,750: *I*_eq_ = 6.85⋅ exp(3.54⋅10^–5^⋅*t*) + 1.097⋅10^–5^⋅exp(2.778⋅10^–3^⋅*t*) ∙ 4,750 < *t* ≤ 5,050: *I*_eq_ = 14.006 ∙ 5,050 < *t* ≤ 8,900: *I*_eq_ = 6.85⋅exp[3.54⋅10^–5^ ⋅(9,800 – *t*)] + 1.097⋅10^–5^⋅exp[2.778⋅10^–3^ ⋅(9,800 – *t*)] ∙ 8,900 < *t* ≤ 10,000: *I*_eq_ = 0
	80% P_0_	∙ 0 < *t* ≤ 5,000: *I*_eq_ = 3.827⋅exp[4.696⋅10^–5^⋅ (1.053⋅*t* + 3,500)] + 6.883⋅10^–7^⋅ exp[1.545⋅10^–3^⋅(1.053⋅*t* + 3,500)] ∙ 5,000 < *t* ≤ 9,250: *I*_eq_ = 3.827⋅exp{4.696⋅10^–5^⋅ [1.053⋅(10,000 – *t*) + 3,500]} + 6.883⋅10^–7^⋅exp {1.545⋅10^–3^⋅[1.053⋅(10,000 – *t*) + 3,500]} ∙ 9,250 < *t* ≤ 10,000: *I*_eq_ = 0	∙ 0 < *t* ≤ 2,000: *I*_eq_ = 8.684⋅exp(-3.416⋅10^–6^⋅*t*) + 0.03⋅exp(0.773⋅10^–3^⋅*t*) ∙ 2,000 < *t* ≤ 5,000: *I*_eq_ = 8.543⋅exp(2.028⋅10^–5^⋅*t*) + 2.749⋅10^–5^⋅exp(2.079⋅10^–3^⋅*t*) - 0.132 ∙ 5,000 < *t* ≤ 8,000: *I*_eq_ = 8.543⋅exp[2.028⋅10^–5^⋅(10,000 – *t*)] + 2.749⋅10^–5^⋅exp [2.079⋅10^–3^⋅(10,000 – *t*)] - 0.132 ∙ 8,000 < *t* ≤ 10,000: *I*_eq_ = 8.684⋅exp[-3.416⋅10^–6^⋅(10,000 – *t*)] + 0.03⋅exp[0.773⋅10^–3^⋅(10,000 – *t*)]	∙ 0 < *t* ≤ 5,000: *I*_eq_ = 6.85⋅exp(1.416⋅10^–5^⋅*t*) + 1.097⋅10^–5^⋅exp(1.111⋅10^–3^⋅*t*) ∙ 5,000 < *t* ≤ 8,800: *I*_eq_ = 6.85⋅exp[1.416⋅10^–5^⋅ (10,000 – *t*)] + 1.097⋅10^–5^⋅exp [1.111⋅10^–3^⋅(10,000 – *t*)] ∙ 8,800 < *t* ≤ 10,000: *I*_eq_ = 0
	60% P_0_	∙ 0 < *t* ≤ 5,000: *I*_eq_ = 3.827⋅exp[4.696⋅10^–5^⋅ (0.59⋅*t* + 3,500)] + 6.883⋅10^–7^⋅ exp[1.545⋅10^–3^⋅ (0.59⋅ *t* + 3,500)] ∙ 5,000 < *t* ≤ 9,250: *I*_eq_ = 3.827⋅exp{4.696⋅10^–5^⋅ [0.59⋅(10,000 – *t*) + 3,500]} + 6.883⋅10^–7^⋅exp {1.545⋅10^–3^⋅[0.59⋅(10,000 – *t*) + 3,500]} ∙ 9,250 < *t* ≤ 10,000: *I*_eq_ = 0	∙ 0 < *t* ≤ 5,000: *I*_eq_ = 8.53⋅exp(-5.754⋅10^–6^⋅*t*) + 0.186⋅exp(2.562⋅10^–4^⋅*t*) ∙ 5,000 < *t* ≤ 10,000: *I*_eq_ = 8.53⋅exp[-5.754⋅10^–6^⋅(10,000 – *t*)] + 0.186⋅exp[2.562⋅10^–4^ ⋅(10,000 – *t*)]	∙ 0 < *t* ≤ 5,000: *I*_eq_ = 6.85⋅ exp(1.77⋅10^–5^⋅0.6⋅*t*) + 1.097⋅10^–5^⋅exp(0.833⋅10^–3^⋅*t*) ∙ 5,000 < *t* ≤ 8,700: *I*_eq_ = 6.85⋅exp[1.062⋅10^–5^⋅ (10,000 – *t*)] + 1.097⋅10^–5^⋅exp [0.833⋅10^–3^⋅ (10,000 – *t*)] ∙ 8,700 < *t* ≤ 10,000: *I*_eq_ = 0
	40% P_0_	∙ 0 < *t* ≤ 5,000: *I*_eq_ = 3.827⋅exp[4.7⋅10^–5^⋅(0.212⋅*t* + 3,500)] + 6.883⋅10^–7^⋅exp[1.545⋅10^–^^3^[*cpsbreak*]⋅(0.212⋅*t* + 3,500)] ∙ 5,000 < *t* ≤ 9,250: *I*_eq_ = 3.827⋅exp{4.696⋅10^–5^⋅[0.212⋅(10,000 – *t*) + 3,500]} + 6.883⋅10^–7^⋅exp{1.545⋅10^–3^ ⋅[0.212⋅(10,000 – *t*) + 3,500]} ∙ 9,250 < *t* ≤ 10,000: *I*_eq_ = 0	∙ 0 < *t* ≤ 10,000: *I*_eq_ = 8.732	∙ 0 < *t* ≤ 5,000: *I*_eq_ = 6.85⋅exp(0.708⋅10^–5^⋅*t*) + 1.097⋅10^–5^⋅exp(0.556⋅10^–3^⋅*t*) ∙ 5,000 < *t* ≤ 8,500: *I*_eq_ = 6.85⋅exp[0.708⋅10^–5^ ⋅(10,000 – *t*)] + 1.097⋅10^–5^⋅exp[0.556⋅10^–3^ ⋅(10,000 – *t*)] ∙ 8,500 < *t* ≤ 10,000: *I*_eq_ = 0
	20% P_0_	∙ 0 < *t* ≤ 262: *I*_eq_ = 6 ∙ 262 < *t* ≤ 10,000: *I*_eq_ = 4.354		∙ 0 < *t* ≤ 8,500: *I*_eq_ = 6.92 ∙ 8,500 < *t* ≤ 10,000: *I*_eq_ = 0
X_m_ = 0 mm	80% P_0_	∙ 0 < *t* ≤ 5,000: *I*_eq_ = 1.166⋅exp[1.058⋅10^–4^⋅(*t* + 5,000)] + 1.033⋅10^–8^⋅exp[1.881⋅10^–3^⋅(*t* + 5,000)] ∙ 5,000 < *t* ≤ 9,100: *I*_eq_ = 1.166⋅exp[1.058⋅10^–4^⋅(15,000 – *t*)] + 1.033⋅10^–8^⋅exp[1.881⋅10^–3^⋅ (15,000 – *t*)] ∙ 9,100 < *t* ≤ 10,000: *I*_eq_ = 0	∙ 0 < *t* ≤ 2,000: *I*_eq_ = 6.249⋅exp(-2.269⋅10^–5^⋅*t*) + 0.519⋅exp(2.798⋅10^–4^⋅*t*) ∙ 2,000 < *t* ≤ 5,000: *I*_eq_ = 6.719⋅exp(1.239⋅10^–5^⋅*t*) + 5.718⋅10^–4^⋅exp(1.709⋅10^–3^⋅*t*) - 0.026 ∙ 5,000 < *t* ≤ 8,000: *I*_eq_ = 6.719⋅exp[1.239⋅10^–5^⋅(10,000 – *t*)] + 5.718⋅10^–4^⋅exp[1.709⋅10^–3^⋅(10,000 – *t*)] - 0.026 ∙ 8,000 < *t* ≤ 10,000: *I*_eq_ = 6.249⋅exp[-2.269⋅10^–5^⋅(10,000 – *t*)] + 0.519⋅exp[2.798⋅10^–4^ ⋅(10,000 – *t*)]	∙ 0 < *t* ≤ 5,000: *I*_eq_ = 6.926⋅exp(3.115⋅10^–5^⋅*t*) + 1.461⋅10^–4^⋅exp(1.903⋅10^–3^⋅*t*) ∙ 5,000 < *t* ≤ 9,000: *I*_eq_ = 6.926⋅exp[3.115⋅10^–5^ ⋅(10,000 – *t*)] + 1.461⋅10^–4^⋅exp[1.903⋅10^–3^ ⋅(10,000 – *t*)] ∙ 9,000 < *t* ≤ 10,000: *I*_eq_ = 0
	60% P_0_	∙ 0 < *t* ≤ 5,000: *I*_eq_ = 1.166⋅exp[1.058⋅10^–4^⋅(0.506⋅*t* + 5,000)] + 1.033⋅10^–8^⋅exp[1.881⋅10^–3^⋅(0.506⋅*t* + 5,000)] ∙ 5,000 < *t* ≤ 9,100: *I*_eq_ = 1.166⋅exp[1.058⋅10^–4^⋅(10,060 - 0.506⋅*t*)] + 1.033⋅10^–8^⋅exp[1.881⋅10^–3^⋅(10,060 - 0.506⋅*t*)] ∙ 9,100 < *t* ≤ 10,000: *I*_eq_ = 0	∙ 0 < *t* ≤ 5,000: *I*_eq_ = 6.554⋅exp(-5.482⋅10^–6^⋅*t*) + 0.213⋅exp(2.105⋅10^–4^⋅*t*) ∙ 5,000 < *t* ≤ 10,000: *I*_eq_ = 6.554⋅exp[-5.482⋅10^–6^⋅(10,000 – *t*)] + 0.213⋅exp[2.105⋅10^–4^⋅(10,000 – *t*)]	∙ 0 < *t* ≤ 5,000: *I*_eq_ = 6.926⋅exp(1.767⋅10^–5^⋅*t*) + 1.461⋅10^–4^⋅exp(1.081⋅10^–3^⋅*t*) ∙ 5,000 < *t* ≤ 9,000: *I*_eq_ = 6.926⋅exp[1.767⋅10^–5^ ⋅(10,000 – *t*)] + 1.461⋅10^–4^⋅exp[1.081⋅10^–3^ ⋅(10,000 – *t*)] ∙ 9,000 < *t* ≤ 10,000: *I*_eq_ = 0
	40% P_0_	∙ 0 < *t* ≤ 8,700: *I*_eq_ = 1.95 ∙ 8,700 < *t* ≤ 10,000: *I*_eq_ = 0	∙ 0 < *t* ≤ 10,000: *I*_eq_ = 6.768	∙ 0 < *t* ≤ 5,000: *I*_eq_ = 6.926⋅exp(0.623⋅10^–5^⋅*t*) + 1.461⋅10^–4^⋅exp(0.381⋅10^–3^⋅*t*) ∙ 5,000 < *t* ≤ 9,000: *I*_eq_ = 6.926⋅exp[0.623⋅10^–5^ ⋅(10,000 – *t*)] + 1.461⋅10^–4^⋅exp[0.381⋅10^–3^ ⋅(10,000 – *t*)] ∙ 9,000 < *t* ≤ 10,000: *I*_eq_ = 0

*The unit of time is a millisecond. ● Indicates individual time intervals and piecewise equations.*

### Muscle Length Variation

The stimulation patterns required for triangular force production by the motor unit during isometric contraction were predicted under three different modes of the muscle length: physiologically minimal (*X*_m_ = −16 mm), optimal (*X*_m_ = −8 mm), and maximal (*X*_m_ = 0 mm) muscle lengths. The physiological range of the variation in muscle length was determined based on the locomotor data of adult cats reported in a previous study (Goslow et al., 1973). The variation in muscle length influences both the motoneuron and muscle unit by transmitting muscle spindle signals to the motoneuron and adjusting a certain kinetic parameter [i.e., K5 in Kim et al. (2015)] of the formation of calcium-binding troponin in the sarcoplasm of the muscle unit model as a function of the muscle length.

### Muscle Afferent Signals

The feedback signals originating from the muscle spindle were simulated by summing the excitatory synaptic inputs (i.e., *G*_esyn_) over both the soma and dendrite (Segev et al., 1990). Under passive membrane conditions, the peak conductance for muscle afferent inputs was determined to match the current (i.e., *I*_N__,PASS_) experimentally measured during voltage clamping over the soma under a 10-mV hyperpolarized potential below the resting potential while slowly varying the muscle length from the physiologically minimum length to the maximum length (Lee et al., 2003). The peak conductance for muscle afferent inputs was set to 0 mS/cm^2^ to yield an *I*_N__,PASS_ value of 0 nA at −16 mm, 0.0064 mS/cm^2^ to yield an *I*_N__,PASS_ value of 2.5 nA at −8 mm, and 0.0128 mS/cm^2^ to yield an *I*_N__,PASS_ value of 5 nA at 0 mm under isometric conditions.

### Simulations

All simulations were performed in the PyMUS software environment (version 2.0.1) *via* the Python integration method (a Variable-coefficient Ordinary Differential Equation (VODE) was applied to the motoneuron, and the Livermore Solver for Ordinary Differential Equations (LSODE) was applied to the muscle unit) at a fixed time step (0.1 ms) on a desktop computer operated by 64-bit Microsoft Windows 10. Default values of the model parameters and simulation conditions were implemented unless noted otherwise in the text. The computer codes of the models and simulations in this study are presented in the supplementary information (Supplementary File 2) and are publicly available from the public repository of GitHub^1^. The data employed for the estimation of the stimulation waveforms required for force production by the motor unit at the various speeds and levels in the present study are presented in the supplementary material (Supplementary File 3).

## Results

### Non-linear Input–Output Relationship of the Motoneuron and Muscle Unit

We first evaluated whether the model motor unit captures the non-linearity of the input–output function of the motoneuron and muscle unit. Figure 2 shows the non-linear response of the motoneuron and muscle unit under linearly increasing and decreasing excitation effects. The model motoneuron suitably replicated experimental observations of non-linear firing of spinal motoneurons during triangular current injection at the cell body [please refer to Figure 3 in Hounsgaard et al. (1984)]. In particular, the firing rate was enhanced at a current intensity higher than the recruitment threshold in the ascending stimulation phase, and firing was sustained below the recruitment threshold in the descending stimulation phase (as shown in the top and middle panels of Figure 2A). This result was mainly attributed to the activation of persistent inward current (PIC)-generating calcium channels at the dendrite underlying the non-linear input–output relationship of the motoneuron (as shown in the bottom panel of Figure 2A). In terms of the model muscle unit, a non-linear relationship of the stimulation frequency and force genesis has been experimentally verified during electrical stimulation of soleus muscles in adult cat preparations [please refer to Figure 8 in Rack and Westbury (1969)]. The muscle unit model reproduced the sigmoidal shape of force production well when slowly increasing and decreasing the stimulation rate (as shown in the top and middle panels, respectively, of Figure 2B). This was mainly attributed to the non-linear dynamics of muscle activation (as shown in the bottom panel of Figure 2B). Furthermore, the non-linear input–output relationship of the motoneuron and muscle unit was modulated by the variation in muscle length, indicating the influence of the muscle afferent inputs and the length dependence of muscle activation. These results indicate that the stimulation waveforms required for the motoneuron to control the muscle force may strongly depend on the muscle length.

### Continuous Stimulation Waveforms for Force Control at the Various Muscle Lengths

**FIGURE 3:**
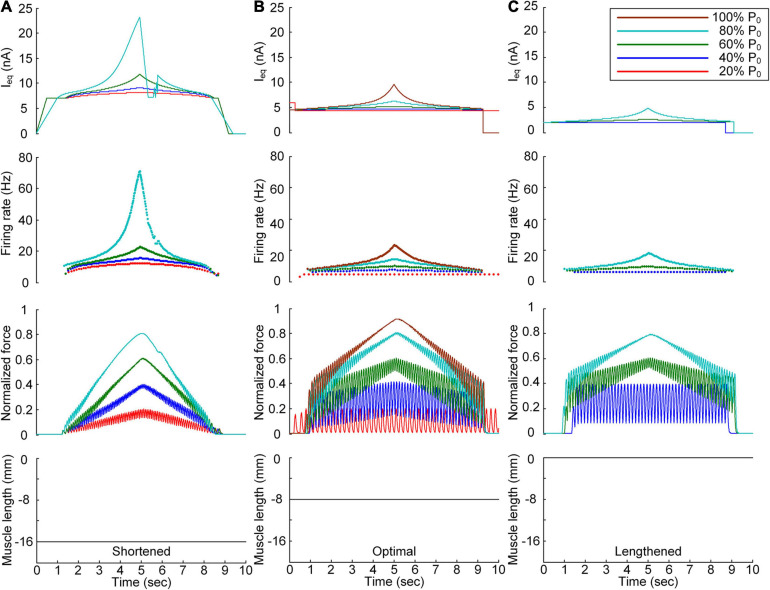
Continuous stimulation waveforms for linear force production. Intensities of continuous current stimulation (I_eq_) applied to the somatic compartment (*top*), instantaneous firing rate (*upper middle*) in the motoneuron, and normalized muscle force (*lower middle*) produced by the muscle unit at the three muscle lengths (*bottom*): physiologically minimum **(A)**, optimal **(B)**, and maximum muscle lengths **(C)**. The level of force production produced by the motor unit is denoted as a percentage of the maximal isometric force (P_0_) at the optimal muscle length and indicated with different colors.

Figure 3 shows the waveforms under continuous current stimulation in regard to linear force generation at the various speeds and levels during isometric contraction at the minimal, optimal, and maximal muscle lengths. Overall, the current intensity exponentially increased and decreased in regard to linear force development and relaxation, respectively, at all muscle lengths. The current stimulation amplitude increased with increasing force speed and magnitude and with decreasing muscle length. The length dependence of the stimulation amplitude was attributed to the reduction in muscle afferent inputs and muscle activation level with decreasing muscle length from the physiologically maximal length. In accordance, the peak firing rate of the motoneuron was the highest at the highest speed and largest magnitude of force production in the shortened muscle state.

In terms of the shortened muscle, the motor unit produced various force levels ranging from 20 to 80% of the maximal force (i.e., P_0_) generated at the optimal muscle length (Figure 3A). The 100% P_0_ level was not achievable due to the bell-shaped length-tension muscle properties, indicating a force decline with decreasing or increasing muscle length from the optimal length. The stimulation waveforms were almost symmetric up to 60% of P_0_. At 80% of P_0_, however, the stimulation intensity rapidly decreased near the recruitment threshold to achieve linear force relaxation in the descending stimulation phase. This was required to prevent the full activation of the PIC channels located across the motoneuron dendrite in the descending stimulation phase. At the optimal muscle length, the current threshold for firing initiation was lower than that in the shortened muscle case due to the excitatory muscle afferent inputs to the motoneuron (Figure 3B). Brief injection of an excitatory step current at the beginning of current stimulation was required to slowly produce force up to 20% of P_0_. This result indicates that the PIC may partially be activated and slowly oscillate, leading to slow firing at the motoneuron. Notably, the model motor unit could not fully reach the maximal force level due to the limitation of the stimulation intensity to prevent full PIC activation in the motoneuron. Once the PIC was fully activated in the ascending stimulation phase, the deactivation of PIC channels became uncontrollable, resulting in difficulty in achieving linear force relaxation in the descending stimulation phase. This result could be attributed to the severe attenuation of the electrical signals generated near the cell body when transmitted to distal dendritic areas in the motoneuron. In terms of the lengthened muscle, the current threshold for firing initiation further decreased due to the increase in excitatory muscle afferent inputs to the motoneuron (Figure 3C). Force production was realized from the peak level of 40% of P_0_ to 80% of P_0_ owing to the twitch force greater than 20% of P_0_ and the bell-shaped length-tension muscle properties.

All these results suggest that the exponential waveform under a continuous current intensity effectively controlled linear force production and that the continuous type of current stimulation could limit the range of force generation in shorter-than-optimal muscles.

### Discrete Stimulation Waveforms for Force Control at the Various Muscle Lengths

**FIGURE 4:**
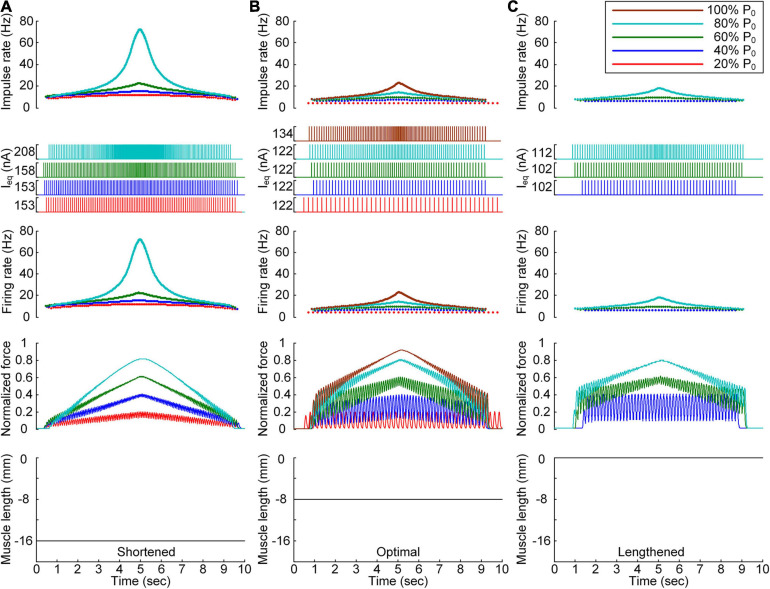
Discrete stimulation waveforms for linear force production. Frequency and amplitude (two *top panels*) of impulse current stimulation (*I*_eq_) applied to the somatic compartment, instantaneous firing rate (*upper middle*) in the motoneuron, and normalized muscle force (*lower middle*) produced by the muscle unit at the three muscle lengths (*bottom*): physiologically minimum **(A)**, optimal **(B)**, and maximum muscle lengths **(C)**. The level of force production produced by the motor unit is denoted as a percentage of the maximal isometric force (P_0_) at the optimal muscle length and indicated with different colors.

After stimulation waveform estimation under continuous stimulation conditions, the stimulation waveforms required for force production were assessed under discrete stimulation conditions (Figure 4). In general, the rate of current impulse stimulation should exponentially increase and then decrease to produce forces according to a triangular shape at all muscle lengths. Similar to the continuous stimulation case, the amplitude and peak rate of the current impulses increased with increasing speed and magnitude of force production and with decreasing muscle length. The length dependence of the stimulation amplitude and rate was attributed to the increase in muscle afferent inputs and muscle activation level with increasing muscle length from the shortened state. In accordance, the peak firing rate of the motoneuron was the highest at the minimal muscle length and the highest speed and level of force production. However, in contrast to the continuous stimulation case, the stimulation waveforms were almost symmetric at all force production levels during isometric contraction at the minimal muscle length (see Figure 4A). Furthermore, the model motor unit fully produced the maximum force at the optimal muscle length without full activation of the PIC channels across the motoneuron (Supplementary File 4).

All these results indicate that the exponential waveform due to the current impulse frequency is effective in regard to linear production of the muscle force and that the discrete type of current stimulation is suitable for the full range of force generation and muscle length levels.

### Influence of the PICs Over the Motoneuron Dendrites on the Stimulation Waveforms

**FIGURE 5:**
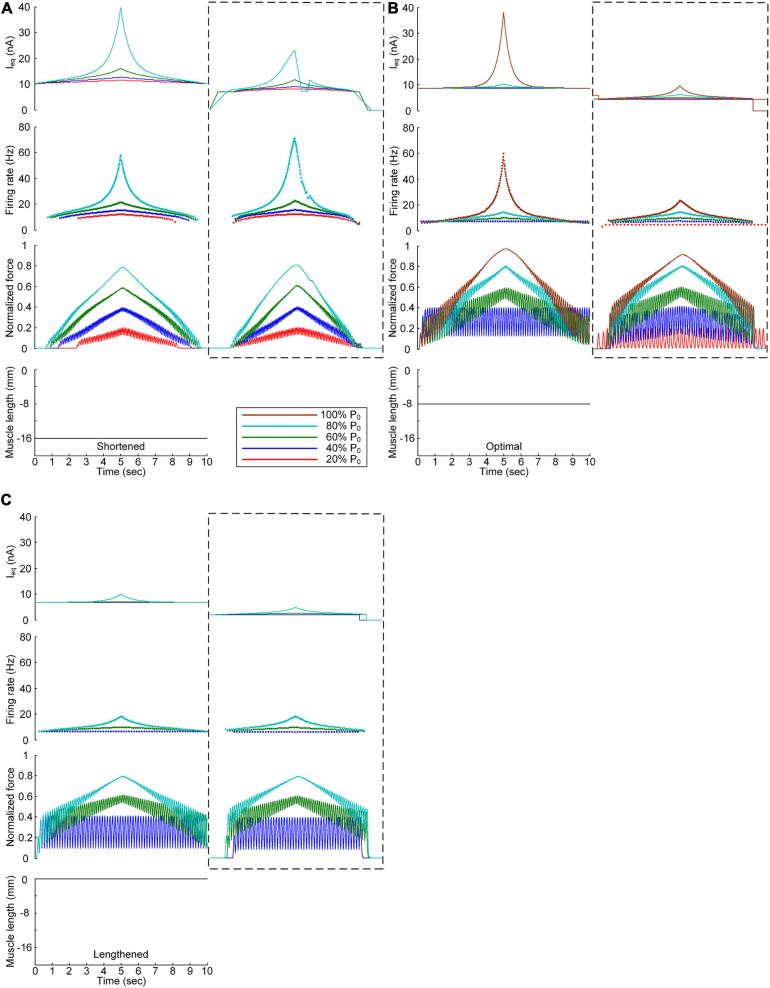
Influence of the PIC on the stimulation waveforms under the continuous conditions. Intensities of continuous current stimulation (*I*_eq_) applied to the somatic compartment (*top*), instantaneous firing rate (*upper middle*) in the motoneuron, and normalized muscle force (*lower middle*) generated by the muscle unit at the three muscle lengths (*bottom*): physiologically minimum **(A)**, optimal **(B)**, and maximum muscle lengths **(C)**. The level of force production produced by the motor unit is denoted as a percentage of the maximal isometric force (P_0_) at the optimal muscle length and indicated with different colors. The dashed-line rectangles indicate the simulation results with the motoneuron PIC channels, as shown in Figure 3, for the purpose of comparison.

We further investigated the influence of the PIC channels responsible for the plateau potentials over the motoneuron dendrites on the stimulation waveforms required for force control. To this end, we compared the stimulation waveforms obtained with and without PIC channels over the dendritic compartment of the motoneuron model, as shown in Figures 3, 4, respectively. Figure 5 shows the stimulation waveforms under continuous stimulation conditions without PIC channels over the motoneuron. Overall, the current stimulation amplitude increased to compensate for the lack of an intrinsic PIC current in the motoneuron when the motoneuron PICs were excluded at all muscle lengths. In contrast to the case involving PIC channels over the motoneuron dendrite (please refer to the dashed-line boxes in Figure 5), the stimulation waveforms were symmetric between the ascending and descending force production phases at all muscle lengths (Figure 5A). Notably, the model motor unit without PIC channels over the motoneuron fully reached the maximal force level at the optimal muscle length during linear force development (Figure 5B). However, the force level of 20% of P_0_ was unattainable because of the increase in the recruitment threshold and firing rate in the absence of motoneuron PIC channels (Figure 5B).

The comparison analysis results revealed the causal relationship of PIC saturation over the motoneuron dendrites and the current intensity limitation and stimulation waveform disruption observed under continuous stimulation conditions.

**FIGURE 6:**
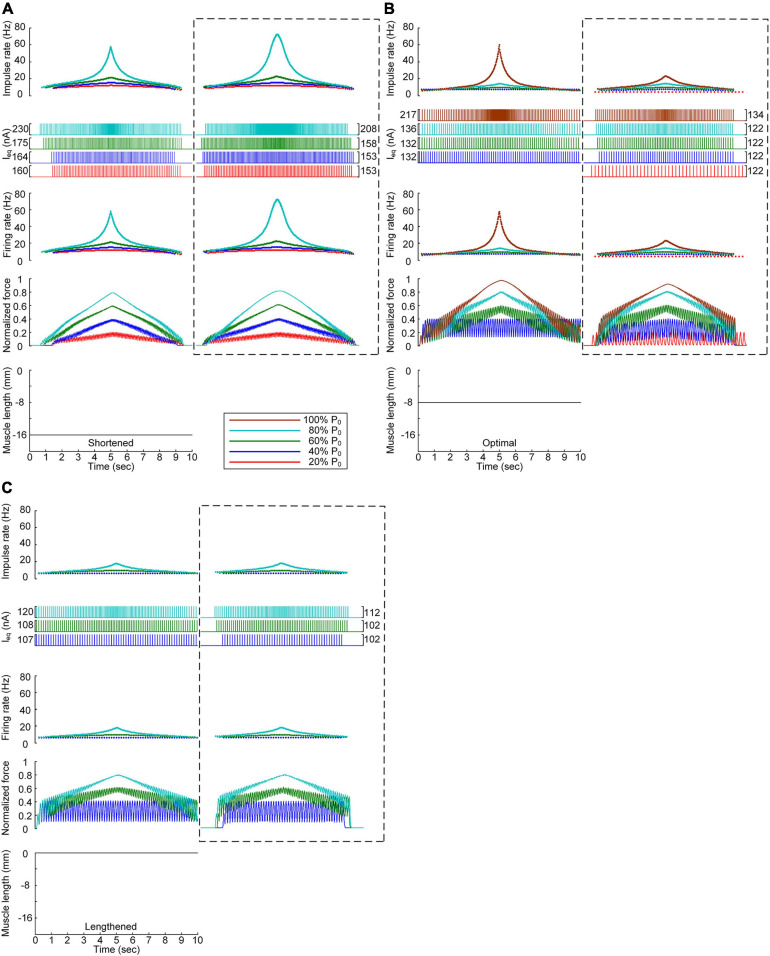
Influence of the PIC on the stimulation waveforms under discrete conditions. Frequency and amplitude (two *top panels*) of impulse current stimulation (I_eq_) applied to the somatic compartment, instantaneous firing rate (*upper middle*) in the motoneuron, and normalized muscle force (*lower middle*) produced by the muscle unit at the three muscle lengths (*bottom*): physiologically minimum **(A)**, optimal **(B)**, and maximum muscle lengths **(C)**. The level of force production by the motor unit is denoted as a percentage of the maximal isometric force (P_0_) at the optimal muscle length and indicated with different colors. The dashed-line rectangles indicate the simulation results with the motoneuron PIC channels, as shown in Figure 4, for the purpose of comparison.

Figure 6 shows the stimulation waveforms under discrete stimulation conditions without PIC channels over the motoneuron dendrites. Overall, the stimulation waveforms were comparable to those in the case with PIC channels over the motoneuron dendrites. The stimulation rate first exponentially increased and then decreased during linear force development and relaxation within the full range (i.e., from 20 to 100% of P_0_). However, the amplitude of the current impulses increased to compensate for the absence of an intrinsic PIC current in the motoneuron. The amplitude increment increased with increasing force level at all muscle lengths (please refer to the dashed-line boxes in Figure 6 representing the PIC activation case). It should be noted that under discrete current stimulation, the model motor unit produced 20% of P_0_ (Supplementary File 5), in addition to the maximal force at the optimal muscle length in the absence of PIC channels over the motoneuron dendrites.

These results reinforce the robustness of discrete stimulation under short-width current pulses for muscle force control purposes regardless of the presence of PICs over the motoneuron dendrites.

### Influence of the Muscle Afferent Inputs on the Stimulation Waveforms

**FIGURE 7:**
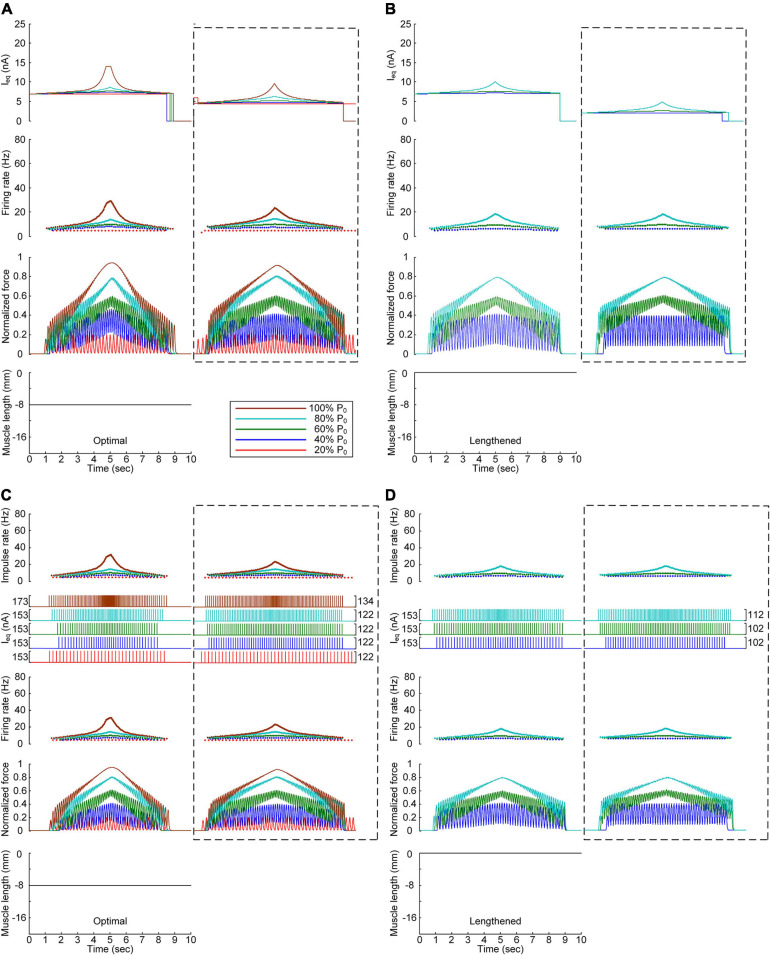
Influence of muscle spindle feedback on the stimulation waveforms. **(A,B)** Intensities of continuous current stimulation (*I*_eq_) applied to the somatic compartment (*top*), instantaneous firing rate (*upper middle*) in the motoneuron, and normalized muscle force (*lower middle*) produced by the muscle unit at the two muscle lengths (*bottom*): physiologically optimal and maximum muscle lengths. **(C,D)** Frequency and amplitude (two *top panels*) of impulse current stimulation (I_eq_) applied to the somatic compartment, instantaneous firing rate (*upper middle*) in the motoneuron, and normalized muscle force (*lower middle*) generated by the muscle unit at the two muscle lengths (*bottom*): physiologically optimal and maximum muscle lengths. The level of force production produced by the motor unit is denoted as a percentage of the maximal isometric force (P_0_) at the optimal muscle length and indicated with different colors. The dashed-line rectangles indicate the simulation results with the motoneuron PIC channels, as shown in Figures 3, 4 for the purpose of comparison.

We also assessed the influence of muscle afferent feedback on the stimulation waveforms for force speed and magnitude control purposes. Figure 7 shows the stimulation waveforms estimated with and without muscle afferent inputs over the motoneuron model, as shown in Figures 3, 4, respectively. Under both continuous and discrete stimulation conditions, the current stimulation amplitude increased to compensate for the lack of muscle afferent inputs to the motoneuron at the optimal and lengthened muscle lengths. In addition, the peak current intensity under continuous current stimulation conditions was relatively high to prevent the full activation of the PIC channels over the motoneuron in the ascending force production phase. This result could be explained by the absence of muscle afferent-mediated facilitation of PIC activation over the motoneuron dendrites (Figure 2A). However, the model motor unit without muscle afferent feedback did not fully reach the maximal force level at the optimal muscle length under continuous motoneuron current stimulation conditions (Figure 7A). Importantly, in the case of discrete current stimulation, the stimulus rate increased until the maximal force level was reached without the full activation of motoneuron PIC channels (Supplementary File 6).

The comparison analysis results indicate that muscle afferent feedback may exacerbate the limitation of the current intensity to prevent full PIC activation during continuous stimulation, and this limitation may also be avoided under discrete stimulation conditions with short-width current pulses.

## Discussion

We estimated the waveforms under extracellular microstimulation near the initial segment and cell body of a spinal motoneuron that control the speed and level of force production by the motor unit during isometric contraction at the various muscle lengths. In terms of both the continuous and discrete stimulation types, the non-linearity of the stimulation waveform systematically increased with increasing force speed and level and with decreasing muscle length. Furthermore, the presence of neuromodulatory inputs from the brainstem and afferent feedback originating from the muscle spindle reduced the current stimulation intensity for linear force production. These results may provide a template for the design of stimulation waveforms under varying force profile, muscle length, and neuromodulation conditions.

### Continuous Versus Discrete Stimulation Protocols

**FIGURE 8:**
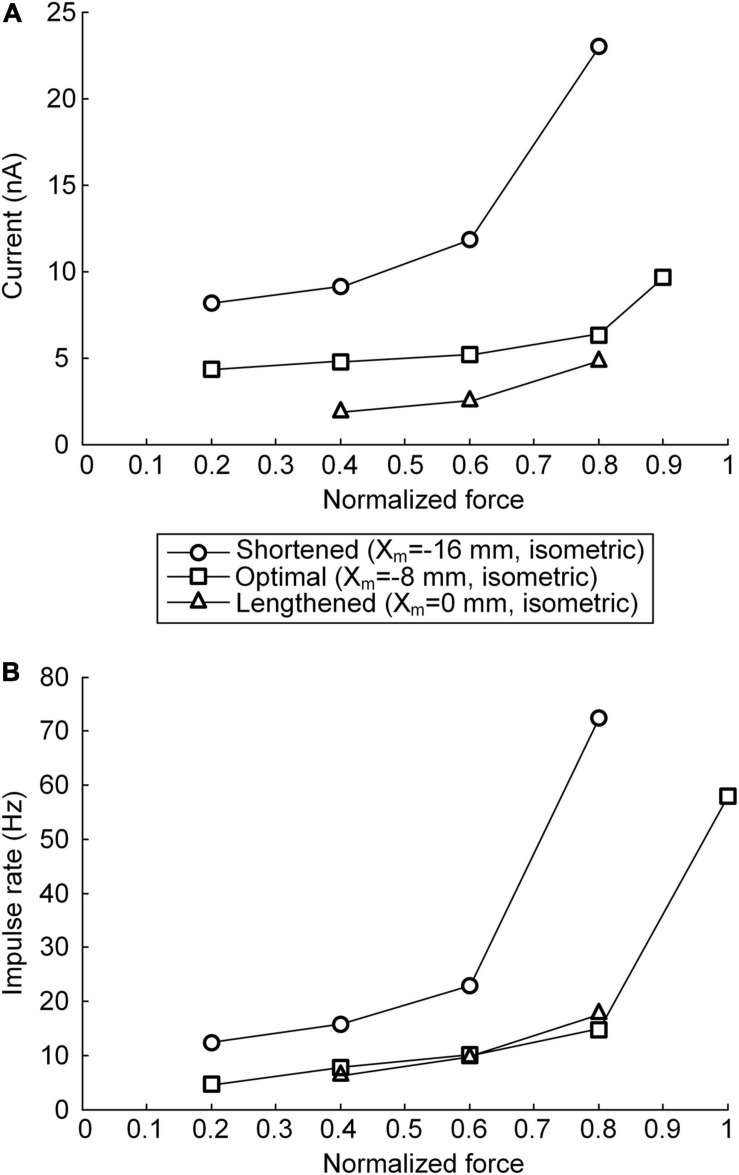
Relationship between current stimulation and force genesis at the various muscle lengths. **(A)** Peak current amplitude eliciting the peak force during triangular force production under continuous stimulation conditions. **(B)** Peak impulse frequency eliciting the peak force during triangular force production under discrete stimulation conditions. The peak force is normalized based on the maximal force isometrically produced at the optimal muscle length. The three symbols (circles, squares, and triangles) indicate the physiological minimal (*X*_m_ = −16 mm), optimal (*X*_m_ = −8 mm), and maximal (*X*_m_ = 0 mm) muscle lengths, respectively.

The non-linearity of the stimulation waveform was assessed by evaluating the difference between the areas under the linear and stimulation curves, i.e., between the lowest and highest points of the stimulation curve (Emancipator and Kroll, 1993). In the present study, the variation in the non-linearity of the stimulation waveform was approximated as the variation in the highest point of the stimulation curve because the lowest point of the stimulation curve remained similar at all produced force levels during isometric contraction at a specific muscle length. Under both the continuous and discrete stimulation protocols, the non-linearity of the current stimulation waveform substantially increased during linear force generation, particularly higher than 60% of the maximal isometric force (i.e., P_0_) at shorter-than-optimal muscle lengths (Figure 8). In addition, the current amplitude increased in the absence of motoneuron PIC channels (please refer to Figures 5, 6) and muscle afferent inputs (Figure 7), indicating the influence of neuromodulation from the brainstem and sensory feedback originating from the muscle on the current stimulation pattern.

The non-linear current stimulation waveforms at the soma of the motoneuron were captured with an exponential function within a broad range of speeds and force production levels at muscle lengths larger than the optimal length (Table 1). In terms of the shortened muscles, however, the stimulation waveforms were considerably distorted to prevent the saturation of motoneuron PICs to achieve linear force relaxation in the descending stimulation phase under continuous stimulation conditions (see Figure 3A). Furthermore, the model motor unit did not fully reach the maximum force at the optimal muscle length without full PIC activation over the motoneuron dendrites in the ascending phase under continuous stimulation (Figure 8A). In contrast, the model motor unit reached all force levels within the full physiological range of the muscle length under discrete stimulation conditions (Figure 8B). This result indicates that at a high level of force generation, continuous current stimulation may sufficiently depolarize the membrane potential of the motoneuron dendrites to fully activate the dendritic PIC channels. However, the activation kinetics of dendritic PIC channels have been reported to be slow (i.e., ∼60 ms) (Carlin et al., 2000). Furthermore, attenuation of the alternating electrical signals tends to be much more severe than that of the direct electrical signals when propagating to the distal dendritic areas in the motoneuron (Kim and Jones, 2012). Thus, discrete current stimulation with a small pulse width (i.e., 0.5 ms) may effectively control the firing output of the motoneuron, thus avoiding the full activation of PIC channels over the motoneuron dendrites.

To this end, under the various conditions of brainstem neuromodulation and muscle length, the discrete stimulation protocol is more suitable than the continuous stimulation protocol for the precise modulation of the muscle force at the motor unit level.

### Comparison to Previous Studies

The motor unit model considered in the present study accurately replicated the non-linear input–output relationship of the motoneuron and muscle unit, as separately investigated in previous experimental studies. The motoneuron model captured the acceleration in firing rate above the recruitment threshold in the ascending phase and the persistence of firing below the recruitment threshold in the descending phase during triangular current injection at the soma in the presence of monoamines (Lee and Heckman, 1998a, 1999). The muscle unit model also reproduced the sigmoidal force production in response to a linear increase and decrease in the stimulation frequency, as experimentally reported in previous studies (Rack and Westbury, 1969; Mrowczynski et al., 2006). The muscle length dependence of the non-linear behavior of the motoneuron and muscle fibers is consistent with the results obtained in a previous computational study involving an anatomically realistic model of the spinal motoneuron (Kim, 2017). Thus, the model motor unit constructed in the PyMUS software environment in this study may provide an efficient computational platform for the estimation of the stimulation waveforms required for force control at the motor unit level under a wide range of physiological conditions, including neuromodulation from the brainstem and sensory feedback originating from the muscle spindle.

To our knowledge, the waveform under intraspinal microstimulation has not yet been systematically investigated for muscle force control under various force profile and muscle length conditions. Seminal work has demonstrated, based on both extracellular and intracellular microelectrodes, that spinal motoneurons may be directly activated at the lowest threshold with a stimulating electrode near the initial segment (Gustafsson and Jankowska, 1976). Recently, a field model of the spinal cord coupled with realistic motoneuron models has been analyzed, suggesting that local cells may be selectively activated without activation of bypassing nerve fibers through asymmetric modulation of the duration and amplitude of the cathodic and anodic phases of the biphasic stimulus current pulse (McIntyre and Grill, 2002). This form of the stimulus current pulse may be applied to the discrete stimulation waveforms estimated in this study to improve the selectivity of the target motoneuron. In addition, the previous study has revealed that the stimulus amplitude must be increased to elicit firing in a one-to-one manner at high stimulation frequencies (McIntyre and Grill, 2002). This phenomenon was also observed in the current study with increasing force level (Figure 4). Furthermore, the motoneuronal output during extracellular microstimulation near the cell body has been evaluated with respect to several factors, including the dendritic active conductance and synaptic contact. The previous analysis has suggested that dendritic active conductance and synaptic contact activation may reduce the current threshold for firing initiation with little effect on the motoneuronal output (McIntyre and Grill, 2002). These results are consistent with those obtained in the present study. The amplitude of the current pulses was lowered with PIC activation over the motoneuron dendrites (Figure 6). The influence of synaptic activation was evaluated by increasing the excitatory synaptic conductance over the somatic compartment of the reduced motoneuron. This increase in the excitatory synaptic conductance reduced the amplitude of the current impulses with little difference observed between the stimulation waveforms (not shown).

The present study was conducted in regard to the direct activation of a spinal motoneuron *via* a microelectrode placed near the initial segment and cell body (Gustafsson and Jankowska, 1976). In contrast, indirect transsynaptic activation of the motoneuron has been verified to mostly occur when the microelectrode is located in dendritic regions likely including PIC channels (Gustafsson and Jankowska, 1976). The aforementioned dendritic location of the excitatory synaptic contacts may greatly facilitate the saturation of dendritic PIC channels, resulting in non-responsiveness of the dendrites to synaptic activation at a low force production level (Heckman et al., 2008). Thus, the effective type and waveform required for extracellular microstimulation might differ in the case of transsynaptic activation of the motoneuron and should be further investigated considering the addition of an additional excitatory synaptic conductance to the dendritic compartment of the reduced motoneuron model constructed in this study.

With the above considerations, the present study extends previous investigations by proposing the effective type (i.e., discrete) and waveform (i.e., exponential) of current stimulation for spinal motoneurons that may lead to the precise control of force production at the motor unit level.

### Potential Contributions to Spinal Cord Modulation

Spinal cord stimulation has been applied to effectively restore complex motor functions through the modulation of neuronal networks in the spinal cord (Harkema et al., 2011; Megia Garcia et al., 2020). Intraspinal microstimulation with a multielectrode array may represent an efficient technique to stimulate and modulate the motor output of targeted motor units for the precise control of muscle forces and voluntary movements. From this perspective, this study may provide a basis to design the type and waveform of current stimulation for the precise control of voluntary muscle contractions. The predictions obtained in this study may be testable with recently developed experimental techniques. The force output of a single motor unit in response to current stimulation intracellularly injected at the soma of a spinal motoneuron has been characterized *via in vivo* mouse preparation (Manuel and Heckman, 2011). In addition, the multielectrode array interface developed for spinal cord stimulation (Meacham et al., 2011) and nerve cuff and flexible split ring electrode developed for selective stimulation of the motor nerve (Deurloo et al., 2003; Lee et al., 2017) could also be employed to test the stimulation waveforms required for force control predicted at the motor unit level in the present study. It should be noted that further analysis is needed for the indirect control of spinal motoneurons through current stimulation *via* the skin on the back (Gogeascoechea et al., 2020) or the epidural portion (Ahmed, 2016) of the spinal cord, which typically involves a variety of interneurons and afferents.

### Modeling Considerations

The fundamental limitations of the reduced modeling approach of spinal motoneurons and muscle fibers have been fully addressed in previous studies (Kim et al., 2014, 2015). To estimate the stimulation pattern more physiologically, several issues should be resolved by improving the current motor unit model in future studies. First, the influence of the dendritic structure and PIC distribution on the motoneuron output should be reflected in the prediction of the stimulation pattern under pathological conditions (Elbasiouny et al., 2010). Second, the active membrane mechanisms over the dendrites of spinal motoneurons should be considered in detail, including calcium-activated potassium currents (Li and Bennett, 2007) and hyperpolarization-activated cation channels (Manuel et al., 2007). Third, the present study was conducted particularly considering slow fatigue-resistible motor units. To evaluate other types of motor units, such as fast fatigable motor units, the stimulation waveforms estimated in this study should be adjusted considering the discrepancy in electrical and mechanical properties, particularly in regard to the somatic input resistance, system time constant, rheobase current, afterhyperpolarization potential, and PIC dynamics in spinal motoneurons (Lee and Heckman, 1999; Heckman and Enoka, 2012), twitch rate and amplitude, progressive force reduction phenomenon (i.e., the sag observed over short contraction time <2 s and fatigue over a long contraction time >2 min), and length– and velocity–tension properties of muscle fibers (Brown et al., 1999; Burke, 2011). Fourth, the present study did not consider the shape of the current pulse (Anderson et al., 2019). Thus, the waveforms predicted in the current study might vary according to the shape of the current pulse. Finally, further investigation is required in terms of the stimulation waveforms to control the force production with the whole muscle, considering the organization of spinal motoneurons exhibiting different anatomical and electrical properties (Zengel et al., 1985; Capogrosso et al., 2013). In addition, the influence of spinal interneurons and peripheral afferents should be considered, particularly in the case of skin or epidural stimulation of the spinal cord (Harkema et al., 2011; Takei and Seki, 2013; Gogeascoechea et al., 2020).

### Concluding Remarks

In principle, the waveform obtained with a microelectrode near the motoneuron cell body to realize isometric force control of the motor unit strongly depends on the stimulation type, neuromodulatory input, and muscle length. Compared to continuous current stimulation, discrete current stimulation more suitably prevents the non-linearities induced by the full activation of PIC-generating channels over the motoneuron dendrites, including muscle spindle feedback. Thus, motor unit function modulation may be more effectively achieved by stimulating spinal motoneurons with short-width current pulses. The model-based approach and waveform equations developed in the present study may provide a basis for the design of stimulation patterns applicable to spinal motoneurons to better understand neuromuscular physiology and neuromodulation of movement disorders *via* a neural interface.

## Data Availability Statement

The original contributions presented in the study are included in the article/Supplementary Material, further inquiries can be directed to the corresponding author.

## Author Contributions

HK conceived and designed the study and wrote the manuscript. YJ and HK performed the simulations and analyzed the data. Both authors contributed to the article and approved the submitted version.

## Conflict of Interest

The authors declare that the research was conducted in the absence of any commercial or financial relationships that could be construed as a potential conflict of interest.

## Footnotes

1 https://github.com/NMSL-DGIST/PyMUS

## Supplementary Material

Supplementary File 1 Equations and descriptions of the model motor unit.

Supplementary File 2 Computer codes of the models and simulations.

Supplementary File 3 Dataset of the current stimulation levels applied to the motoneuron for force production of the muscle unit at the various speeds and levels, as shown in Figures 3–7.

Supplementary File 4 Production of 100% of the maximal force by the model motor unit, as shown in Figure 4, at the optimal muscle length under discrete current stimulation.

Supplementary File 5 Production of 20% of the maximal force by the model motor unit, as shown in Figure 6, at the optimal muscle length under discrete current stimulation conditions.

Supplementary File 6 Production of 100% of the maximal force by the model motor unit, as shown in Figure 7, at the optimal muscle length under discrete current stimulation conditions.

## Abbreviations

VI: voltage–current
IV: current–voltage
VODE: variable-coefficient ordinary differential equation
LSODE: livermore solver for ordinary differential equations
PIC: persistent inward current
PyMUS: python-based motor unit simulator.
