# Supplementary figures and images for: Effective Stimulation Type and Waveform for Force Control of the Motor Unit System: Implications for Intraspinal Microstimulation
Source: Front Neurosci. 2021 Jun 28;15:645984. doi: 10.3389/fnins.2021.645984 (PMC8274570; doi:10.3389/fnins.2021.645984)

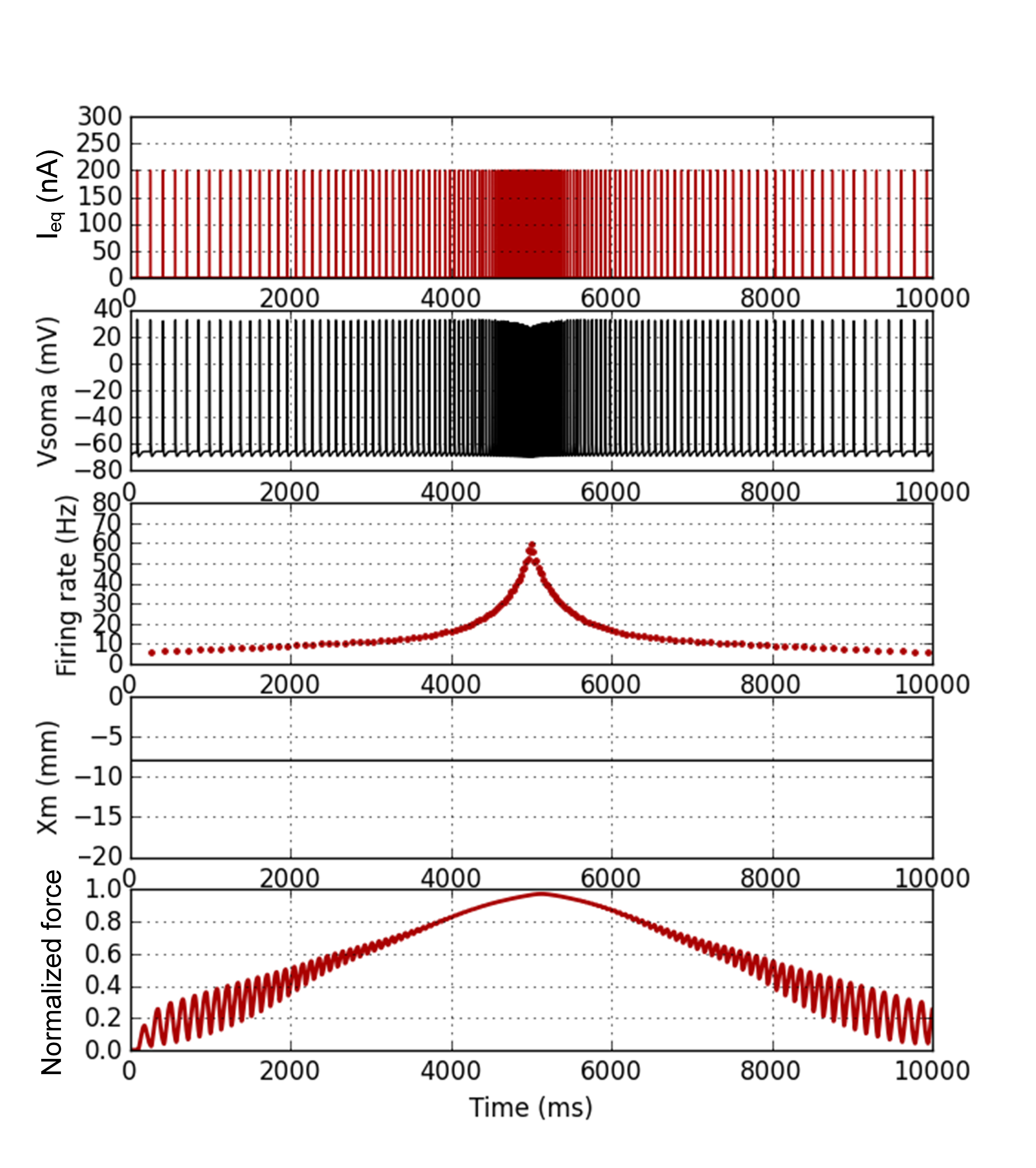

Supplement: Supplementary File 1 — Equations and descriptions of the model motor unit. [file Image_1.TIF]

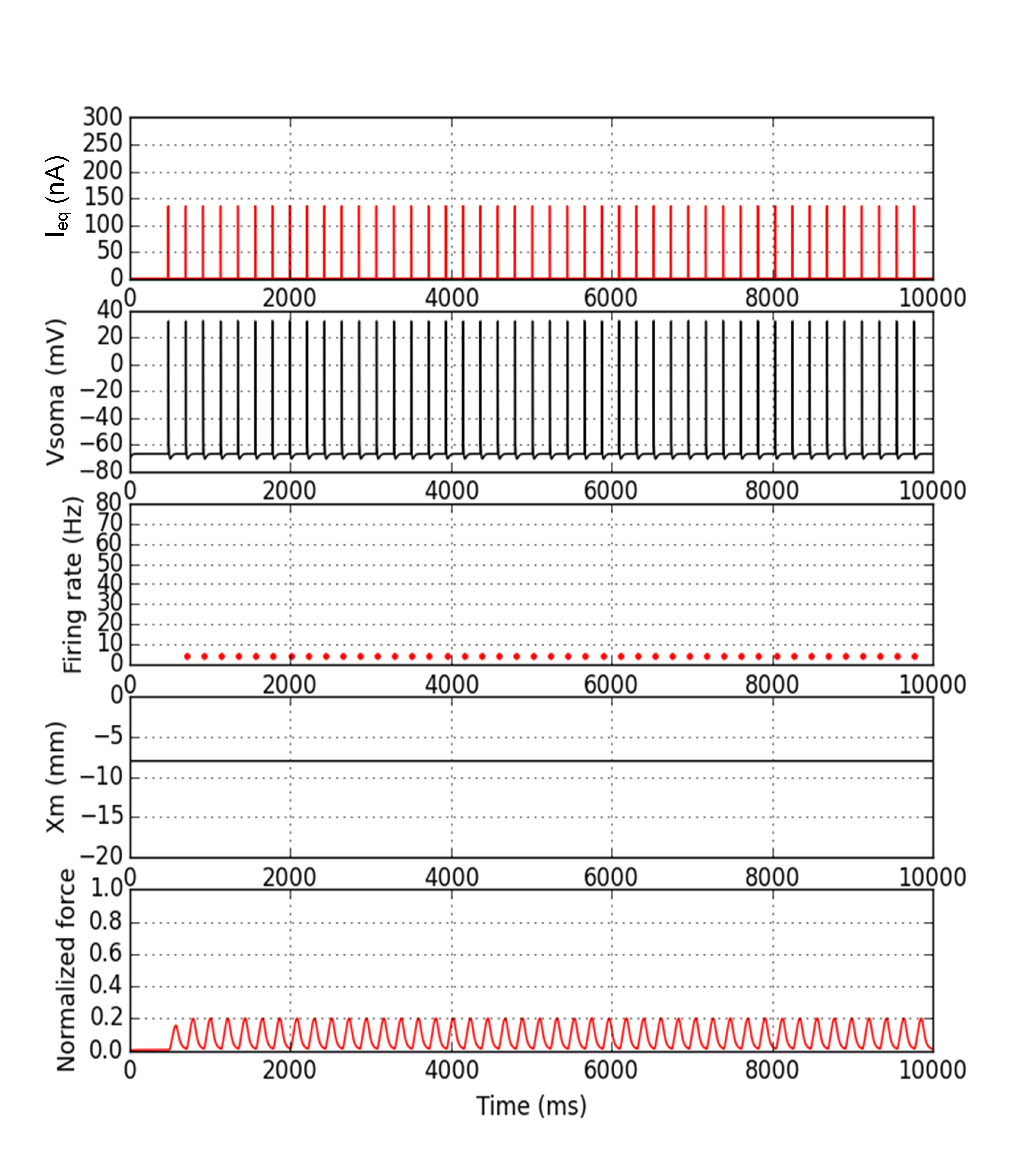

Supplement: Supplementary File 2 — Computer codes of the models and simulations. [file Image_2.TIF]

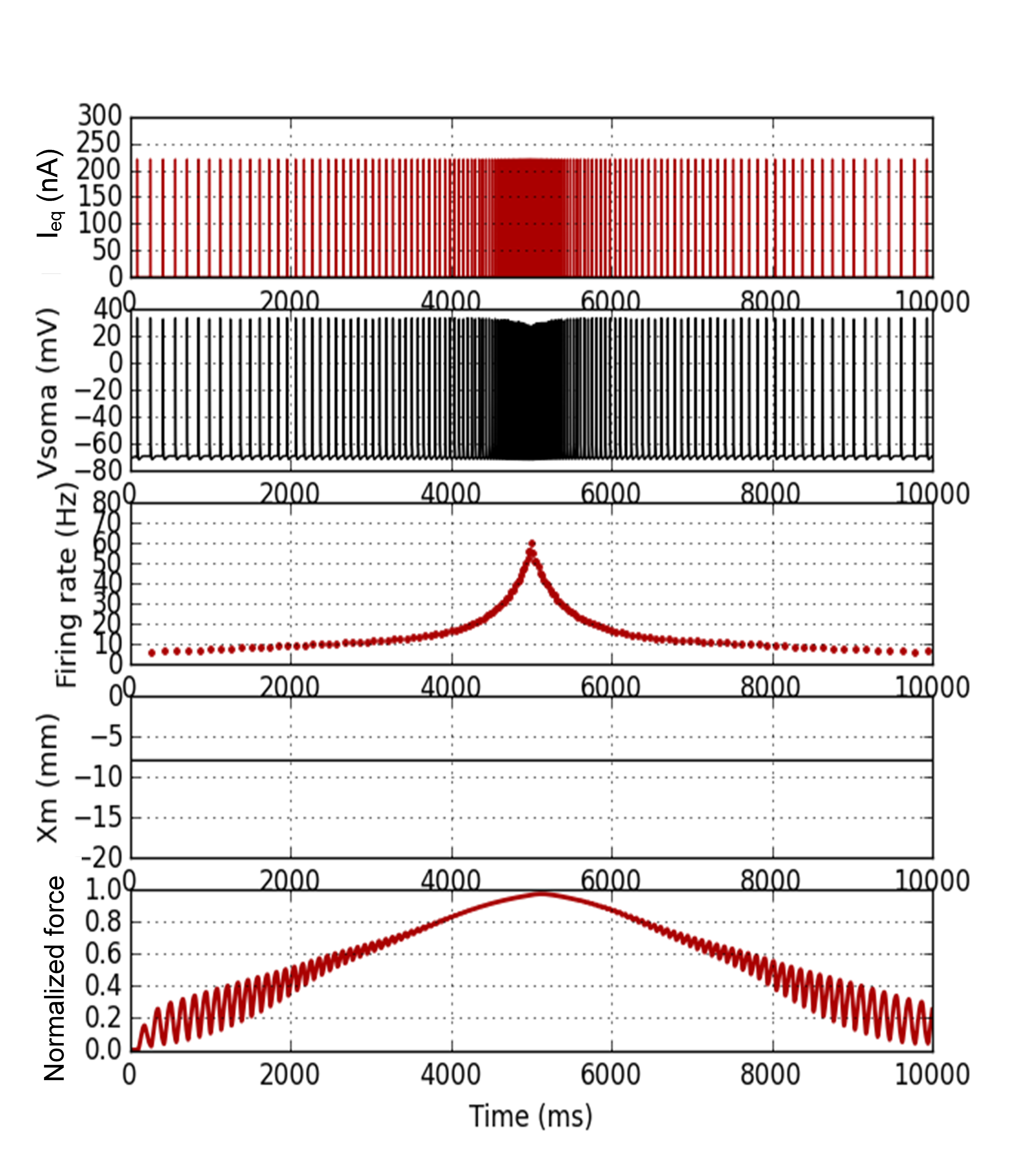

Supplement: Supplementary File 3 — Dataset of the current stimulation levels applied to the motoneuron for force production of the muscle unit at the various speeds and levels, as shown in Figures 3–7. [file Image_3.TIF]

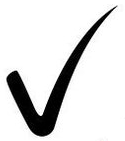

Supplement: Supplementary File 5 — Production of 20% of the maximal force by the model motor unit, as shown in Figure 6, at the optimal muscle length under discrete current stimulation conditions. [file Data_Sheet_2.ZIP › 20201125_PyMUS_ver2.0.1_Release/resources/default.PNG]

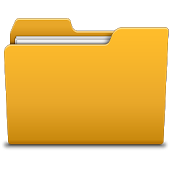

Supplement: Supplementary File 5 — Production of 20% of the maximal force by the model motor unit, as shown in Figure 6, at the optimal muscle length under discrete current stimulation conditions. [file Data_Sheet_2.ZIP › 20201125_PyMUS_ver2.0.1_Release/resources/file_load.png]

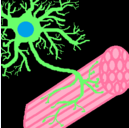

Supplement: Supplementary File 5 — Production of 20% of the maximal force by the model motor unit, as shown in Figure 6, at the optimal muscle length under discrete current stimulation conditions. [file Data_Sheet_2.ZIP › 20201125_PyMUS_ver2.0.1_Release/resources/PyMUS_image.png]
