# Supplementary material for: Effective Stimulation Type and Waveform for Force Control of the Motor Unit System: Implications for Intraspinal Microstimulation
Source: Front Neurosci. 2021 Jun 28;15:645984. doi: 10.3389/fnins.2021.645984 (PMC8274570; doi:10.3389/fnins.2021.645984)
Supplement: Supplementary File 4 — Production of 100% of the maximal force by the model motor unit, as shown in Figure 4, at the optimal muscle length under discrete current stimulation. [file Data_Sheet_1.PDF]

The modeling procedure, including the equation derivation and parameter setting for the motoneuron and muscle fibers, was fully presented in our previous studies (Kim et al., 2014;Kim et al., 2015). Default parameter values set for a cat slow-type motor unit in the PyMUS software (version 2.0.1) were applied to the present study.

1 System equations used for the reduced motoneuron model

$$C_{m,S} \cdot \frac{dV_S}{dt} = -\sum I_{soma} - G_{m,S} \cdot \left(V_S - E_{Leak,S}\right) - \frac{G_C}{p} \cdot (V_S - V_D) + I_{eq} \quad (1.1)$$

$$\sum I_{soma} = I_{Naf} + I_{Kdr} + I_{Can} + I_{K(Ca)} + I_{Nap} + I_H + I_{esyn}$$

$$C_{m,D} \cdot \frac{dV_D}{dt} = -\sum I_{dendrite} - G_{m,D} \cdot \left(V_D - E_{Leak,D}\right) - \frac{G_C}{1-p} \cdot (V_D - V_S) \quad (1.2)$$

$$\sum I_{dendrite} = I_{Cal} + I_{esyn}$$

where the subscripts *S* and *D* indicate the soma and dendrites, respectively, *V* is the transmembrane potential, E_{Leak} is the reversal potential of the leak current, *G* and *C* indicate specific membrane conductance and capacitance, $\sum I$ indicates transmembrane currents and I_S is intracellularly injected current at the soma. Both $E_{Leak,S}$ and $E_{Leak,D}$ were set to -70 mV in the present study.

1.1 *Equations for the cable parameters reflecting morphological & electrotonic properties*

$$G_{m,S} = \frac{1 - VA_{DS}^{\ \ DC}}{r_N\,(1 - VA_{SD}^{\ \ DC} VA_{DS}^{\ \ DC})} \quad (1.3)$$

$$G_{m,D} = \frac{pVA_{DS}^{\ \ DC}(1 - VA_{SD}^{\ \ DC})}{(1-p) r_N VA_{SD}^{\ \ DC}(1 - VA_{SD}^{\ \ DC} VA_{DS}^{\ \ DC})} \quad (1.4)$$

$$G_C = \frac{pVA_{DS}^{\ \ DC}}{r_N\,(1 - VA_{SD}^{\ \ DC} VA_{DS}^{\ \ DC})} \quad (1.5)$$

$$C_{m,D} = \frac{1}{\omega(1-p)} \sqrt{\frac{G_C^{\ 2}}{\left(VA_{SD}^{\ \ AC}\right)^2} - \left\{G_C + G_{m,D}(1-p)\right\}^2} \quad (1.6)$$

$$C_{m,S} = \frac{\tau_m \left\{p(1-p)\tau_m G_{m,S} G_{m,D} + pG_{m,S}\left(\tau_m G_C - C_{m,D}\right) + p^2 G_{m,S} C_{m,D} + (1-p)\left(\tau_m G_C G_{m,D} - G_C C_{m,D}\right)\right\}}{p\left\{(1-p)\left(\tau_m G_{m,D} - C_{m,D}\right) + \tau_m G_C\right\}} \quad (1.7)$$

where r_N is the value of somatic input resistance (R_N) normalized to the surface area of the somatic compartment, τ_m is the membrane time constant, *VA* indicates the voltage attenuation factor calculated between the soma and dendrites, and *p* is the ratio of the surface area accumulated up to a specific path length (D_{path}) from the soma to the total surface area. In the current study, R_N, τ_m, VA_{SD}^{DC}, VA_{DS}^{DC}, VA_{SD}^{AC}, *p*, the surface area for the somatic compartment up to the D_{path}=0.6 mm, ω, and conduction velocity (i.e., CV_{axon}) of axonal nerve were set to 1.29 MΩ, 0.0072 sec, 0.76, 0.75, 0.27, 0.492, 0.3157 mm^2, 2π×250 Hz and 100 m/s, respectively.

1.2 *Equations for the active currents*

All voltage gated ion channels were modeled based on the HH type formulation as follows: $I_{Ion}=G_{Ion}\cdot m_{ion}^{a}\cdot h_{ion}^{b}\cdot(V-E_{Ion})$, where G_{Ion} is the peak conductance of the specific ion current, m_{ion} and h_{ion} are the gating variables for activation and inactivation, *a* and *b* are the order of activation and inactivation, and E_{Ion} is the reversal potential for the ion of interest. The active mechanisms included in the soma or the dendrite were indicated by the subscript *S* or *D* in the following equations.

[SOMA]

Ca^{2+} concentration dynamics

$\frac{d[Ca^{2+}]_i}{dt} = f\cdot(-\alpha\cdot I_{Can} - K_{Ca}\cdot[Ca^{2+}]_i)$ where f=0.01, α=1.0 mol/μC/cm^2, K_{Ca}=8.0 ms^{-1}. (1.7)

Equilibrium potential for Ca^{2+}

$E_{Ca} = \frac{1000\cdot R\cdot T}{Z_{Ca}\cdot F}\cdot log\left(\frac{[Ca^{2+}]_o}{[Ca^{2+}]_i}\right) - 70$ where R=8.31441 VC/mol·K, T=309.15 K, Z_{Ca}=2, F=96485.309 C/mol, $[Ca^{2+}]_o$=2.0 mM.

Fast Na$^{\pm}$ current

$I_{Naf} = G_{Naf}\cdot {m_{naf}}^3\cdot h_{naf}\cdot(V_S - E_{Na})$ where G_{Naf}=26.75 mS/cm2, E_{Na}=50.0 mV. (1.8)

$\frac{dm_{naf}}{dt} = \alpha_m\cdot(1-m_{naf}) - \beta_m\cdot m_{naf}$ where $\alpha_m = \frac{\alpha_1\cdot(V_S+\alpha_2)}{exp\left(-\frac{V_S+\alpha_2}{\alpha_3}\right)+\alpha_4}$, $\beta_m = \frac{\beta_1\cdot(V_S-\beta_2)}{exp\left(\frac{V_S-\beta_2}{\beta_3}\right)+\beta_4}$, α_1=-0.4 $(mV\cdot mS)^{-1}$, α_2=-49.0 mV, α_3=5.0 mV, α_4=-1.0, β_1=0.4 $(mV\cdot mS)^{-1}$, β_2=-25.0 mV, β_3=5.0 mV,

β_4=-1.0.

$\frac{dh_{naf}}{dt} = \frac{h_\infty - h_{naf}}{\tau_h}$ where $h_\infty = \frac{1}{1+exp\left(\frac{V_S-\gamma_1}{\gamma_2}\right)}$, $\tau_h = \frac{\gamma_6}{exp\left(\frac{V_S-\gamma_3}{\gamma_4}\right)+exp\left(-\frac{V_S-\gamma_3}{\gamma_5}\right)}$, γ_1=-58.0 mV, γ_2=7.0 mV, γ_3=-60.0 mV, γ_4=15.0 mV, γ_5=16.0 mV, γ_6=30.0 ms.

Persistent Na^+ current:

$I_{Nap} = G_{Nap} \cdot m_{nap}{}^3 \cdot (V_S - E_{Na})$ where G_{Nap}=0.00086 mS/cm2, E_{Na}=50.0 mV. (1.9)

$\frac{d(m_{nap})}{dt} = \alpha_m \cdot (1 - m_{nap}) - \beta_m \cdot m_{nap}$ where $\alpha_m = \frac{\alpha_1 \cdot (V_S-\alpha_2)}{exp\left(-\frac{V_S-\alpha_2}{\alpha_3}\right)+\alpha_4}$, $\beta_m = \frac{\beta_1 \cdot (V_S-\beta_2)}{exp\left(\frac{V_S-\beta_2}{\beta_3}\right)+\beta_4}$, α_1=-

-0.0353 (mV·mS)$^{-1}$, α_2=-21.4 mV, α_3=5.0 mV, α_4=-1.0, β_1=0.00088 (mV·mS)$^{-1}$, β_2=-25.7 mV, β_3=5.0 mV, β_4=-1.0.

Delayed rectifier K^+ current

$I_{Kdr} = G_{Kdr} \cdot n_{kdr}{}^4 \cdot (V_S - E_K)$ where G_{Kdr}=6.2 mS/cm^2, E_K=-80.0 mV. (1.10)

$\frac{d(n_{kdr})}{dt} = \frac{(n_\infty - n_{kdr})}{\tau_n}$ where $n_\infty = \frac{1}{1+exp\left(-\frac{V_S-\gamma_1}{\gamma_2}\right)}$, $\tau_n = \frac{\gamma_6}{exp\left(\frac{V_S-\gamma_3}{\gamma_4}\right)+exp\left(-\frac{V_S-\gamma_3}{\gamma_5}\right)}$, γ_1=-31.0 mV, γ_2=15.0 mV, γ_3=-50.0 mV, γ_4=40.0 mV, γ_5=50.0 mV, γ_6=5.0 ms.

Calcium dependent K+ current

$I_{K(Ca)} = G_{K(Ca)} \cdot \frac{[Ca^{2+}]_i}{[Ca^{2+}]_i + K_d} \cdot (V_S - E_K)$ where $G_{K(Ca)}$=0.54 mS/cm^2, E_K=-80.0 mV, K_d=0.0005 mM. (1.11)

N-Type Ca^{2+} current

$I_{Can} = G_{Can} \cdot m_{can}{}^2 \cdot h_{can} \cdot (V_S - E_{Ca})$ where G_{Can}=0.008 mS/cm^2. (1.12)

$\frac{d(m_{can})}{dt} = \frac{m_\infty - m_{can}}{\tau_m}$ where $m_\infty = \frac{1}{1+exp\left(-\frac{V_S-\gamma_1}{\gamma_2}\right)}$, $\tau_m = \gamma_3$, γ_1=-25.0 mV, γ_2=5.0 mV, γ_3=15.0 ms.

$\frac{d(h_{can})}{dt} = \frac{h_\infty - h_{can}}{\tau_h}$ where $h_\infty = \frac{1}{1+exp\left(\frac{V_S-\gamma_1}{\gamma_2}\right)}$, $\tau_h = \gamma_3$, γ_1=-43.0 mV, γ_2=5.0 mV, γ_3=50.0 ms.

Synaptic current

$I_{esyn} = G_{esyn} \cdot (V_S - E_{esyn})$ where G_{esyn}= 0 for x_m=-16 mm, G_{esyn}=0.0064 mS/cm^2 for x_m=-8

mm, G_{esyn}=0.0128 mS/cm^2 for x_m=0 mm, E_{esyn}=0.0 mV. (1.13)

[DENDRITE]

Equilibrium potential for Ca^{2+}

$E_{Ca} = 60\ mV$ (constant calcium reversal potential) (1.14)

Low voltage activation L-type Ca^{2+} current

$I_{Cal} = G_{Cal} \cdot l_{cal} \cdot (V_D - E_{Ca})$ where G_{Cal}=0.124 mS/cm^2. (1.15)

$\frac{dl_{cal}}{dt} = \frac{l_\infty - l_{cal}}{\tau_l}$ where $l_\infty = \frac{1}{1+exp\left(-\frac{V_D-\gamma_1}{\gamma_2}\right)}$, $\tau_l = \gamma_3$, γ_1=-43.0 mV, γ_2=6.0 mV, γ_3=60.0 ms.

Synaptic current

$I_{esyn} = G_{esyn} \cdot \left(V_D - E_{esyn}\right)$ where G_{esyn}= 0 for x_m=-16 mm, G_{esyn}=0.0064 mS/cm^2 for x_m=-8 mm, G_{esyn}=0.0128 mS/cm^2 for x_m=0 mm, E_{esyn}=0.0 mV. (1.16)

2 System equations used for the muscle-tendon model

Module 1: The transformation of action potentials to calcium dynamics in the sarcoplasm

$$\frac{d[Ca_{SR}]}{dt} = -K1 \cdot CS_0 \cdot [Ca_{SR}] + (K1 \cdot [Ca_{SR}] + K2) \cdot [Ca_{SR}CS] - R + U \quad (2.1)$$

$$\frac{d[Ca_{SR}CS]}{dt} = K1 \cdot CS_0 \cdot [Ca_{SR}] - (K1 \cdot [Ca_{SR}] + K2) \cdot [Ca_{SR}CS] \quad (2.2)$$

where $[Ca_{SR}]$, $[Ca_{SR}CS]$ and CS_0 indicate the concentration of free calcium ions, Ca^{2+} bound to calsequestrin and total calsequestrin in the sarcoplasmic reticulum (*SR*), respectively, *K1* and *K2* are the forward and backward constants for reaction kinetics between the Ca_{SR} and $Ca_{SR}CS$ and the release (*R*) of Ca^{2+} from the SR and the uptake (*U*) of Ca^{2+} into the SR were mathematically modeled as

$$R = [Ca_{SR}] \cdot R_{\max} \cdot \sum_{i=1}^{n} \left(1 - \exp\left(-\frac{t-t_i}{\tau_1}\right)\right) \cdot \exp\left(-\frac{t-t_i}{\tau_2}\right),$$

$$U = U_{\max} \cdot \left(\frac{[Ca_{SP}]^2 \cdot K^2}{1 + [Ca_{SP}] \cdot K + [Ca_{SP}]^2 \cdot K^2}\right)^2$$

$$\frac{d[Ca_{SP}]}{dt} = -(K3 \cdot B_0 + K5 \cdot T_0) \cdot [Ca_{SP}] + (K3 \cdot [Ca_{SP}] + K4) \cdot [Ca_{SP}B] + (K5 \cdot [Ca_{SP}] + K6) \cdot [Ca_{SP}T] + R - U \quad (2.3)$$

$$\frac{d[Ca_{SP}B]}{dt} = K3 \cdot B_0 \cdot [Ca_{SP}] - (K3 \cdot [Ca_{SP}] + K4) \cdot [Ca_{SP}B] \quad (2.4)$$

$$\frac{d[Ca_{SP}T]}{dt} = K5 \cdot T_0 \cdot [Ca_{SP}] - (K5 \cdot [Ca_{SP}] + K6) \cdot [Ca_{SP}T] \quad (2.5)$$

where [Ca_{SP}], [$Ca_{SP}B$], [$Ca_{SP}T$], B_0 and T_0 indicate the concentration of free calcium ions, Ca^{2+} bound to free calcium-buffering proteins (B), Ca^{2+} bound to troponin (T), total free calcium-buffering proteins and total troponin in the sarcoplasm (SP), respectively and $K3$-$K6$ are the rate constants for chemical reactions between the Ca_{SP}, B, T, $Ca_{SP}B$ and $Ca_{SP}T$ in which $K5$ and $K6$ were modulated as a function of muscle length (X_m) and activation level ($\tilde{A}$) under steady Ca^{2+} stimulation as follows,

$$K5 = K5_i \cdot \varphi(X_m), \begin{cases} \varphi(X_m) = \varphi_1 \cdot X_m + \varphi_2, & for\, X_m < optimal\, length \\ \varphi(X_m) = \varphi_3 \cdot X_m + \varphi_4, & for\, X_m \geq optimal\, length \end{cases},$$

$$K6 = \frac{K6_i}{1 + 5 \cdot \tilde{A}}$$

In the current study, $K1$, $K2$, $K3$, $K4$, $K5_i$, $K6_i$, K, R_{max}, U_{max}, τ_1, τ_2, φ_1, φ_2, φ_3, φ_4, CS_0, B_0 and T_0 were set to 3000.0 $M^{-1}\cdot ms^{-1}$, 3.0 ms^{-1}, 400.0 $M^{-1}\cdot ms^{-1}$, 1.0 ms^{-1}, 400000.0 $M^{-1}\cdot ms^{-1}$, 150.0 ms^{-1}, 850.0 M^{-1}, 10.0 ms^{-1}, 2000.0 $M\cdot ms^{-1}$, 3.0 ms, 25.0 ms, 0.03 mm^{-1}, 1.23, 0.01 mm^{-1}, 1.08, 30 mM, 0.43 mM and 70 μM, respectively.

<u>Module 2: The transformation of the sarcoplasmic calcium dynamics to muscle activation dynamics</u>

$$A(t) = (\tilde{A})^{\alpha} \quad (2.6)$$

where the $\tilde{A}(t)$ was mathematically modeled as

$$\frac{d\tilde{A}}{dt} = \frac{\tilde{A}_\infty - \tilde{A}}{\tau_{\tilde{A}}} \text{ where } \tilde{A}_\infty = 0.5 \cdot \left(1 + \tanh \frac{[Ca_{SP}T]/T_0 - C1}{C2}\right), \; \tau_{\tilde{A}} = C3 \cdot \left(\cosh \frac{[Ca_{SP}T]/T_0 - C4}{2 \cdot C5}\right)^{-1}$$

In the current study, α, $C1$, $C2$, $C3$, $C4$ and $C5$ were set to 2, 0.128, 0.093, 61.206 ms, -13.116 and 5.095, respectively.

Module 3: The transformation of muscle activation to muscle force

$$F = P_0 \cdot K_{SE} \cdot (\Delta X_m - \Delta X_{CE}) \quad (2.7)$$

where P_0 is the peak force at the optimal length under full excitation in the isometric condition, K_{SE} is the stiffness of the serial element normalized by P_0 and the length (X_{CE}) of contractile element was calculated using the modified Hill-Mashma equations along with the length-tension relationship ($g(X_m)$),

$$\frac{dX_{CE}}{dt} = \frac{-b_0 \cdot \left(P_0 \cdot g(X_m) \cdot A(t) - F\right)}{F + a_0 \cdot g(X_m) \cdot A(t)}, \quad for\ F \le P_0 \cdot g(X_m) \cdot A(t)$$

$$\frac{dX_{CE}}{dt} = \frac{-d_0 \cdot \left(P_0 \cdot g(X_m) \cdot A(t) - F\right)}{2 \cdot P_0 \cdot g(X_m) \cdot A(t) - F + c_0 \cdot g(X_m) \cdot A(t)}, \quad for\ F > P_0 \cdot g(X_m) \cdot A(t)$$

$$g(X_m) = \exp\left\{-\left(\frac{X_m - g_1}{g_2}\right)^2\right\}$$

In the current study, K_{SE}, P_0, g_1, g_2, a_0, b_0, c_0 and d_0 were set to 0.4 mm^{-1}, 1 N, -8.0 mm, 21.4 mm, 0.102 N, 24.35 $mm \cdot s^{-1}$, -0.322 N and 30.3 $mm \cdot s^{-1}$, respectively.

3 References

[1] Kim, H., Jones, K.E., and Heckman, C.J. (2014). Asymmetry in signal propagation between the soma and dendrites plays a key role in determining dendritic excitability in motoneurons. *PLoS One* 9, e95454.

[2] Kim, H., Sandercock, T.G., and Heckman, C.J. (2015). An action potential-driven model of soleus muscle activation dynamics for locomotor-like movements. *J Neural Eng* 12, 046025.
