# Supplementary material for: Effective Stimulation Type and Waveform for Force Control of the Motor Unit System: Implications for Intraspinal Microstimulation
Source: Front Neurosci. 2021 Jun 28;15:645984. doi: 10.3389/fnins.2021.645984 (PMC8274570; doi:10.3389/fnins.2021.645984)
Supplement: Supplementary File 5 — Production of 20% of the maximal force by the model motor unit, as shown in Figure 6, at the optimal muscle length under discrete current stimulation conditions. [file Data_Sheet_2.ZIP › 20201125_PyMUS_ver2.0.1_Release/model_equations.pdf]

The modeling procedure, including the equation derivation and parameter setting for the motoneuron and muscle fibers, was fully presented in our previous studies (Kim et al., 2014;Kim et al., 2015).

1. System equations used for the reduced motoneuron model

$$C_{m,S} \cdot \frac{dV_S}{dt} = -\sum I_{soma} - G_{m,S} \cdot (V_S - E_{Leak,S}) - \frac{G_C}{p} \cdot (V_S - V_D) + I_S \tag{1.1}$$

$$\sum I_{soma} = I_{Naf,S} + I_{Kdr,S} + I_{Can,S} + I_{K(Ca),S} + I_{Nap,S} + I_{H,S} + I_{syn,S}$$

$$C_{m,D} \cdot \frac{dV_D}{dt} = -\sum I_{dendrite} - G_{m,D} \cdot (V_D - E_{Leak,D}) - \frac{G_C}{1-p} \cdot (V_D - V_S) \tag{1.2}$$

$$\sum I_{dendrite} = I_{Naf,D} + I_{Kdr,D} + I_{Can,D} + I_{K(Ca),D} + I_{Nap,D} + I_{H,D} + I_{Cal,D} + I_{syn,D}$$

where the subscripts *S* and *D* indicate the soma and dendrites, respectively, *V* is the membrane potential, E_{Leak} is the reversal potential of the leak current, *G* and *C* indicate specific membrane conductance and capacitance, $\sum I$ indicates transmembrane currents and I_S is intracellularly injected current at the soma.

1.1 *Inverse equations for the five cable parameters*

$$G_{m,S} = \frac{1 - VA_{DS}{}^{DC}}{r_N (1 - VA_{SD}{}^{DC} VA_{DS}{}^{DC})} \tag{1.3}$$

$$G_{m,D} = \frac{p VA_{DS}{}^{DC} (1 - VA_{SD}{}^{DC})}{(1-p) r_N VA_{SD}{}^{DC} (1 - VA_{SD}{}^{DC} VA_{DS}{}^{DC})} \tag{1.4}$$

$$G_C = \frac{p VA_{DS}{}^{DC}}{r_N (1 - VA_{SD}{}^{DC} VA_{DS}{}^{DC})} \tag{1.5}$$

$$C_{m,D} = \frac{1}{\omega(1-p)} \sqrt{\frac{G_C{}^2}{\left(VA_{SD}{}^{AC}\right)^2} - \{G_C + G_{m,D}(1-p)\}^2} \tag{1.6}$$

$$C_{m,S} = \frac{\tau_m \{p(1-p)\tau_m G_{m,S} G_{m,D} + p G_{m,S}(\tau_m G_C - C_{m,D}) + p^2 G_{m,S} C_{m,D} + (1-p)(\tau_m G_C G_{m,D} - G_C C_{m,D})\}}{p\{(1-p)(\tau_m G_{m,D} - C_{m,D}) + \tau_m G_C\}} \tag{1.7}$$

where *VA* indicates the voltage attenuation factor calculated between the soma and dendrites and r_N is the value of R_N normalized to the surface area of the somatic compartment. In the current version of PyMUS (PyMUS v2.0), the surface area for the somatic compartment, *p* and *ω* were set to 0.3157 m^2, 0.5 and 2π×250 Hz, respectively.

1.2 *Equations for the active currents*

In the current version of PyMUS (PyMUS v2.0), the soma and dendrite have the same types of active membrane properties, except for the additional inclusion of low voltage activated L-type calcium current in the dendrite. All voltage gated ion channels were modeled based on the HH type formulation as follows: $I_{Ion}=G_{Ion} \cdot m_{ion}{}^a \cdot h_{ion}{}^b \cdot (V-E_{Ion})$, where G_{Ion} is the peak conductance of the specific ion current, m_{ion} and h_{ion} are the gating variables for activation and inactivation, *a* and *b* are the order of activation and inactivation, and E_{Ion} is the reversal potential for the ion of interest. The active mechanisms included in both the soma and the dendrite were indicated by the subscript *X* unless otherwise stated with the subscript *S* or *D* in the following equations.

Fast Na$^{\pm}$ current

$$I_{Naf,X} = G_{Naf,X} \cdot m_{naf}{}^{3} \cdot h_{naf} \cdot \left(V_X - E_{Na,X}\right) \tag{1.8}$$

$$\frac{dm_{naf}}{dt} = \alpha_m \cdot \left(1 - m_{naf}\right) - \beta_m \cdot m_{naf} \text{ where}$$

$$\alpha_m = \frac{\alpha_{nafm1,X} \cdot \left(V_X + \alpha_{nafm2,X}\right)}{\exp\left(-\frac{V_X + \alpha_{nafm2,X}}{\alpha_{nafm3,X}}\right) + \alpha_{nafm4,X}}, \quad \beta_m = \frac{\beta_{nafm1,X} \cdot \left(V_X - \beta_{nafm2,X}\right)}{\exp\left(\frac{V_X - \beta_{nafm2,X}}{\beta_{nafm3,X}}\right) + \beta_{nafm4,X}}$$

$$\frac{dh_{naf}}{dt} = \frac{h_\infty - h_{naf}}{\tau_h} \text{ where}$$

$$h_\infty = \frac{1}{1 + \exp\left(\frac{V_X - \gamma_{nafh1,X}}{\gamma_{nafh2,X}}\right)}, \quad \tau_h = \frac{\gamma_{nafh6,X}}{\exp\left(\frac{V_S - \gamma_{nafh3,X}}{\gamma_{nafh4,X}}\right) + \exp\left(-\frac{V_S - \gamma_{nafh3,X}}{\gamma_{nafh5,X}}\right)}$$

Delayed rectifier K^{+} current

$$I_{Kdr,X} = G_{Kdr,X} \cdot n_{kdr}{}^{4} \cdot (V_X - E_{K,X}) \tag{1.9}$$

$$\frac{d(n_{kdr})}{dt} = \frac{(n_\infty - n_{kdr})}{\tau_n} \text{ where}$$

$$n_\infty = \frac{1}{1 + \exp\left(-\frac{V_X - \gamma_{kdrm1,X}}{\gamma_{kdrm2,X}}\right)}, \quad \tau_n = \frac{\gamma_{kdrm6,X}}{\exp\left(\frac{V_X - \gamma_{kdrm3,X}}{\gamma_{kdrm4,X}}\right) + \exp\left(-\frac{V_X - \gamma_{kdrm3,X}}{\gamma_{kdrm5,X}}\right)}$$

N-Type Ca^{2+} current

$$I_{Can,X} = G_{Can,X} \cdot m_{can}{}^{2} \cdot h_{can} \cdot (V_X - E_{Ca,X}) \tag{1.10}$$

$$\frac{d(m_{can})}{dt} = \frac{m_\infty - m_{can}}{\tau_m} \text{ where } m_\infty = \frac{1}{1 + \exp\left(-\frac{V_X - \gamma_{canm1,X}}{\gamma_{canm2,X}}\right)}, \quad \tau_m = \gamma_{canm3,X}$$

$$\frac{d(h_{can})}{dt} = \frac{h_\infty - h_{can}}{\tau_h} \text{ where } h_\infty = \frac{1}{1 + \exp\left(\frac{V_X - \gamma_{canh1,X}}{\gamma_{canh2,X}}\right)}, \quad \tau_h = \gamma_{canh3,X}$$

Ca^{2+} concentration dynamics

$$\frac{d[Ca^{2+}]_{i,X}}{dt} = f_X(-\alpha_X \cdot I_{Can,X} - K_{Ca,X} \cdot [Ca^{2+}]_{i,X}) \tag{1.11}$$

Equilibrium potential for Ca^{2+}

$$E_{Ca,X} = \frac{1000 \cdot R \cdot T}{Z_{Ca} \cdot F} \cdot \log\left(\frac{[Ca^{2+}]_{o,X}}{[Ca^{2+}]_{i,X}}\right) - 70 \tag{1.12}$$

where R=8.31441 VC/mol·K, T=309.15 K, Z_{Ca}=2, F=96485.309 C/mol with $[Ca^{2+}]_o$=2 mM.

Calcium dependent K+ current

$$I_{K(Ca),X} = G_{K(Ca),X} \cdot \frac{[Ca^{2+}]_{i,X}}{[Ca^{2+}]_{i,X} + K_{d,X}} \cdot (V_X - E_{K,X}) \tag{1.13}$$

Persistent Na^+ current:

$$I_{Nap,X} = G_{Nap,X} \cdot m_{nap}{}^3 \cdot (V_X - E_{Na,X}) \tag{1.14}$$

$$\frac{d(m_{nap})}{dt} = \alpha_m \cdot (1 - m_{nap}) - \beta_m \cdot m_{nap} \text{ where}$$

$$\alpha_m = \frac{\alpha_{napm1,X} \cdot (V_X - \alpha_{napm2,X})}{\exp\left(-\frac{V_X - \alpha_{napm2,X}}{\alpha_{napm3,X}}\right) + \alpha_{napm4,X}}, \quad \beta_m = \frac{\beta_{napm1,X} \cdot (V_X - \beta_{napm2,X})}{\exp\left(\frac{V_X - \beta_{napm2,X}}{\beta_{napm3,X}}\right) + \beta_{napm4,X}}$$

Hyperpolarization-activated mixed cation current

$$I_{H,X} = G_{H,X} \cdot m_h \cdot (V_X - E_{H,X}) \tag{1.15}$$

$$\frac{d(m_h)}{dt} = \frac{m_\infty - m_h}{\tau_m} \text{ where } m_\infty = \frac{1}{\exp\left(\frac{V_s + \gamma_{hm1,X}}{\gamma_{hm2,X}}\right)}, \quad \tau_m = \gamma_{hm3,X}$$

Synaptic current

$$I_{syn,X} = I_{esyn,X} + I_{isyn,X} \tag{1.16}$$

$$I_{esyn,X} = G_{esyn,X} \cdot (V_X - E_{esyn,X})$$

$$\frac{dG_{esyn,X}}{dt} = -\frac{1}{\tau_{esyn,X}} \cdot (G_{esyn,X} - G_{esyn0,X}) + \sqrt{\frac{2\sigma_{esyn,X}{}^2}{\tau_{esyn,X}}} \cdot \chi_1$$

$$I_{isyn,X} = G_{isyn,X} \cdot (V_X - E_{isyn,X})$$

$$\frac{dG_{isyn,X}}{dt} = -\frac{1}{\tau_{isyn,X}} \cdot (G_{isyn,X} - G_{isyn0,X}) + \sqrt{\frac{2\sigma_{isyn,X}{}^2}{\tau_{isyn,X}}} \cdot \chi_2$$

Dynamical variation in excitatory ($G_{esyn,X}$) and inhibitory ($G_{isyn,X}$) synaptic conductance with noise was formulated using the Ornstein-Uhlenbeck process, where $G_{esyn0,X}$ and $G_{isyn0,X}$ are the mean of $G_{esyn,X}$ and $G_{isyn,X}$, τ is a time constant, σ is the standard deviation from the mean conductance and χ is a random Gaussian noise process with a mean of 0 and a standard deviation of 1 (Destexhe et al., 2001).

Low voltage activation L-type Ca^{2+} current

$$I_{Cal,D} = G_{Cal,D} \cdot l_{cal} \cdot (V_D - E_{Ca,D}) \tag{1.17}$$

$$\frac{dl_{cal}}{dt} = \frac{l_\infty - l_{cal}}{\tau_l} \text{ where } l_\infty = \frac{1}{1 + \exp\left(-\frac{V_D - \gamma_{calm1,D}}{\gamma_{calm2,D}}\right)}, \quad \tau_l = \gamma_{calm3,D}$$

2. System equations used for the muscle-tendon model

Module 1: The transformation of action potentials to calcium dynamics in the sarcoplasm

$$\frac{d[Ca_{SR}]}{dt} = -K1 \cdot CS_0 \cdot [Ca_{SR}] + (K1 \cdot [Ca_{SR}] + K2) \cdot [Ca_{SR}CS] - R + U \quad (2.1)$$

$$\frac{d[Ca_{SR}CS]}{dt} = K1 \cdot CS_0 \cdot [Ca_{SR}] - (K1 \cdot [Ca_{SR}] + K2) \cdot [Ca_{SR}CS] \quad (2.2)$$

where $[Ca_{SR}]$, $[Ca_{SR}CS]$ and CS_0 indicate the concentration of free calcium ions, Ca^{2+} bound to calsequestrin and total calsequestrin in the sarcoplasmic reticulum (*SR*), respectively, *K1* and *K2* are the forward and backward constants for reaction kinetics between the Ca_{SR} and $Ca_{SR}CS$ and the release (*R*) of Ca^{2+} from the SR and the uptake (*U*) of Ca^{2+} into the SR were mathematically modeled as

$$R = [Ca_{SR}] \cdot R_{\max} \cdot \sum_{i=1}^{n} \left(1 - \exp\left(-\frac{t - t_i}{\tau_1}\right)\right) \cdot \exp\left(-\frac{t - t_i}{\tau_2}\right),$$

$$U = U_{\max} \cdot \left(\frac{[Ca_{SP}]^2 \cdot K^2}{1 + [Ca_{SP}] \cdot K + [Ca_{SP}]^2 \cdot K^2}\right)^2$$

$$\frac{d[Ca_{SP}]}{dt} = -(K3 \cdot B_0 + K5 \cdot T_0) \cdot [Ca_{SP}] + (K3 \cdot [Ca_{SP}] + K4) \cdot [Ca_{SP}B] + (K5 \cdot [Ca_{SP}] + K6) \cdot [Ca_{SP}T] + R - U \quad (2.3)$$

$$\frac{d[Ca_{SP}B]}{dt} = K3 \cdot B_0 \cdot [Ca_{SP}] - (K3 \cdot [Ca_{SP}] + K4) \cdot [Ca_{SP}B] \quad (2.4)$$

$$\frac{d[Ca_{SP}T]}{dt} = K5 \cdot T_0 \cdot [Ca_{SP}] - (K5 \cdot [Ca_{SP}] + K6) \cdot [Ca_{SP}T] \quad (2.5)$$

where $[Ca_{SP}]$, $[Ca_{SP}B]$, $[Ca_{SP}T]$, B_0 and T_0 indicate the concentration of free calcium ions, Ca^{2+} bound to free calcium-buffering proteins (*B*), Ca^{2+} bound to troponin (*T*), total free calcium-buffering proteins and total troponin in the sarcoplasm (*SP*), respectively and *K3*-*K6* are the rate constants for chemical reactions between the Ca_{SP}, *B*, *T*, $Ca_{SP}B$ and $Ca_{SP}T$ in which *K5* and *K6* were modulated as a function of muscle length (X_m) and activation level ($\tilde{A}$) under steady Ca^{2+} stimulation as follows,

$$K5 = K5_i \cdot \varphi(X_m), \begin{cases} \varphi(X_m) = \varphi_1 \cdot X_m + \varphi_2, & for\ X_m < optimal\ length \\ \varphi(X_m) = \varphi_3 \cdot X_m + \varphi_4, & for\ X_m \geq optimal\ length \end{cases},$$

$$K6 = \frac{K6_i}{1 + 5 \cdot \tilde{A}}$$

In the current version of PyMUS (PyMUS v2.0), CS_0, B_0 and T_0 were set to 30 (mM), 0.43 (mM) and 70 (µM), respectively.

Module 2: The transformation of the sarcoplasmic calcium dynamics to muscle activation dynamics

$$A(t) = \frac{\left(\tilde{A}\right)^{\alpha(t)}}{\left(1+\beta\cdot\varphi(X_m)\right)\cdot\left(1+\gamma\cdot V_m\right)} \tag{2.6}$$

where V_m is the time derivative of X_m and the $\tilde{A}(t)$ and its exponent ($\alpha(t)$) were mathematically modeled as

$$\frac{d\tilde{A}}{dt} = \frac{\tilde{A}_\infty - \tilde{A}}{\tau_{\tilde{A}}} \text{ where } \tilde{A}_\infty = 0.5\cdot\left(1+\tanh\frac{[Ca_{SP}T]/T_0 - C1}{C2}\right),\ \tau_{\tilde{A}} = C3\cdot\left(\cosh\frac{[Ca_{SP}T]/T_0 - C4}{2\cdot C5}\right)^{-1}$$

$$\alpha(t) = \begin{cases} \alpha \ \textit{for isometric and isokinetic contraction} \\ \alpha + \alpha_1\cdot\left(1+\tanh\frac{t-\alpha_2}{\alpha_3}\right) \textit{for dynamic contraction} \end{cases}$$

In the current version of PyMUS (PyMUS v2.0), α and α_1– α_3 were set to 2, 4.77, 400 (ms) and 160 (ms), respectively. The β and γ were set to 0.47 and 0.001 (s/mm) for lengths shorter than the optimal length during muscle lengthening under dynamic contraction condition otherwise both were set to 0.

<u>Module 3: The transformation of muscle activation to muscle force</u>

$$F = P_0 \cdot K_{SE} \cdot (\Delta X_m - \Delta X_{CE}) \tag{2.7}$$

where P_0 is the peak force at the optimal length under full excitation in the isometric condition, K_{SE} is the stiffness of the serial element normalized by P_0 and the length (X_{CE}) of contractile element was calculated using the modified Hill-Mashma equations along with the length-tension relationship ($g(X_m)$),

$$\frac{dX_{CE}}{dt} = \frac{-b_0\cdot\left(P_0\cdot g(X_m)\cdot A(t) - F\right)}{F + a_0\cdot g(X_m)\cdot A(t)},\ \textit{for } F \le P_0\cdot g(X_m)\cdot A(t)$$

$$\frac{dX_{CE}}{dt} = \frac{-d_0\cdot\left(P_0\cdot g(X_m)\cdot A(t) - F\right)}{2\cdot P_0\cdot g(X_m)\cdot A(t) - F + c_0\cdot g(X_m)\cdot A(t)},\ \textit{for } F > P_0\cdot g(X_m)\cdot A(t)$$

$$g(X_m) = \exp\left\{-\left(\frac{X_m - g_1}{g_2}\right)^2\right\}$$

In the current version of PyMUS (PyMUS v2.0), a_0 and c_0 determined from P_0, length-tension and velocity-tension relationship for entire muscle were scaled by multiplying the ratio of new to default P_0 for simulations of muscle units that may show various levels of P_0.

References

[1] Destexhe, A., Rudolph, M., Fellous, J.M., and Sejnowski, T.J. (2001). Fluctuating synaptic conductances recreate in vivo-like activity in neocortical neurons. *Neuroscience* 107**,** 13-24.

[2] Kim, H., Jones, K.E., and Heckman, C.J. (2014). Asymmetry in signal propagation between the soma and dendrites plays a key role in determining dendritic excitability in motoneurons. *PLoS One* 9**,** e95454.

[3] Kim, H., Sandercock, T.G., and Heckman, C.J. (2015). An action potential-driven model of soleus muscle activation dynamics for locomotor-like movements. *J Neural Eng* 12**,** 046025.
