# Supplementary material for: Effective Stimulation Type and Waveform for Force Control of the Motor Unit System: Implications for Intraspinal Microstimulation
Source: Front Neurosci. 2021 Jun 28;15:645984. doi: 10.3389/fnins.2021.645984 (PMC8274570; doi:10.3389/fnins.2021.645984)
Supplement: Supplementary File 5 — Production of 20% of the maximal force by the model motor unit, as shown in Figure 6, at the optimal muscle length under discrete current stimulation conditions. [file Data_Sheet_2.ZIP › 20201125_PyMUS_ver2.0.1_Release/README.docx]

The PyMUS software can be launched by running the script file of GUI.py in the open-source Python software environment (Python 2.7) such as Anaconda (version 2.2.0). The following packages are needed to be installed before running the codes.

- Scipy libraries (version 0.15.1) to solve the models.
- Pandas (version 0.15.2) and Numpy (version 1.9.2) libraries for data management.
- PyQt4 (version 4.10.4) and Matplotlib (version 1.4.3) for the GUI design and data plotting
